# An optimized YOLO NAS based framework for realtime object detection

**DOI:** 10.1038/s41598-025-17919-w

**Published:** 2025-09-25

**Authors:** Chhaya Gupta, Nasib Singh Gill, Preeti Gulia, Abhinav Kumar, Hanen Karamti, Demmelash Mollalign Moges

**Affiliations:** 1 Department of Computer Science and Applications, Maharshi Dayanand University, Rohtak, India; 2 Faculty at School of Information Technology, Vivekananda Institute of Professional Studies-TC, Delhi, India; 3 Centre for Research Impact and Outcome, Chitkara University Institute of Engineering and Technology, Chitkara University, Rajpura, 140401 Punjab India; 4 Department of Mechanical Engineering and Renewable Energy, Technical Engineering College, The Islamic University, Najaf, Iraq; 5 Department of Computer Sciences, College of Computer and Information Sciences, Princess Nourah bint Abdulrahman University, P.O. Box 84428, Riyadh, 11671 Saudi Arabia; 6 Department of Mathematics, Hawassa University, P.O. Box 05, Hawassa, Ethiopia

**Keywords:** Artificial bee colony (ABC), MISH activation function, Computer vision, Deep learning, Neural network, Real-time object recognition, YOLO-NAS, Medical research, Engineering

## Abstract

An enhanced version of the YOLO-NAS object detection network model has been presented in this paper, and MISH activation and Artificial Bee Colony (ABC) optimization algorithms are integrated. MISH functional adds non-monotonic behavior, which at the same time enhances the feature representation and complements the gradient flow. ABC optimization that assists in the optimization of the hyperparameters to a ground truth and resistance to the models. The given model is tested on the dataset that is introduced by the researchers themselves, and it shows better results compared to baselines based on the YOLO-NAS variants in precision, recall, and mean average precision (mAP) measures. Experiments prove the fact that a combination of a biologically inspired optimizer and a contemporary activation function helps to make training more stable and predictions more accurate. The results show that the proposed fine-tuned YOLO-NAS model outperformed the other tested models, that is, YOLOv6, YOLOv7, and YOLOv8, in the three metrics of accuracy, recall, precision, F1 score, and mAP at 0.50, 0.75, and 0.95 on the test dataset. The proposed model achieved an accuracy of 98% while recognizing real-time objects.

## Introduction

Computer vision has become more of the essence of artificial intelligence (AI), as machines become brighter and more precise in reading and interpreting visual data. The effect that it has extends to many practical areas, including visual perception automation in many fields, which is necessary to enhance efficiency, safety, and decision-making. Computer vision is transformative in healthcare in the field of medical diagnostics and is transformative because it allows the early and accurate diagnosis of, and treatment of, diseases via medical imaging technologies like X-rays, CT, and MRI^1^. It aids in clinical decisions and helps in sophisticated tasks such as robotic surgeries and segmentation of tumors. Intelligent vision systems have found application in the field of monitoring, anomaly detection, facial recognition, and behavior analysis of people in the domain of surveillance and security, which has helped control crime and ensure the security of people. Computer vision technologies have brought about the advancement of autonomous vehicles, traffic flow management systems, and advanced driver-assistance systems in the field of transportation, and they contribute to the reduced number of accidents and enhanced mobility in smart cities. On the same note, computer vision is also transforming precision farming in agriculture, where farmers can know the health of their crops, detect weeds, identify pests, and estimate yield levels, among others, through automation to ensure food security and sustainable agricultural operations^2^.

With the ever-growing range of applications of computer vision, the evaluation of high-precision, low-latency, resource-conserving object detection systems has grown in importance^3^. Occlusions, different lighting conditions, and background clutter are frequent problems in real-world scenarios, and there are cases of small or barely visible objects. Object detection systems capable of long-term deployment in resource-constrained scenarios such as mobile platforms, drones, and edge computing devices should be accompanied by a robust framework that not only instills good performance in terms of detection but also makes optimization towards real-time execution possible. This has spurred the development of deep learning-based detection models, and one such model is the YOLO family, which aims at achieving a trade-off between speed and accuracy. This has been progressed by the development of optimized architectures like the YOLO-NAS, but it has more capability based on the advancement of neural architecture search, activation mechanism, and design with a lightweight status that can be applied in real-world applications across different sectors.

Object detection is one of the main components used in computer vision. Object detection identifies a moving or non-stationary item in an image or video clip. When tracking moving things, object detection is the most crucial stage. In addition to categorizing the images, an effort is made to accurately infer the ideas and locations of the items in each image to comprehend them fully. Object detection has several applications, including face^4^, skeleton^5^, pedestrian^6^, weapon^7^, remote-images detection^8^, and many other applications^9,10^. It is a computer-intensive technology connected to computer vision and image processing. It works with sophisticated images and recordings to recognize occurrences of semantic entities of a particular type (like people or cars).

At first, the primary goal was to increase accuracy, which resulted in models that were even more sophisticated. Installation on the edge and mobile devices was not considered^11^. Two-stage detectors work by first identifying areas of the image with a high probability of holding items and then classifying and locating those objects. Single-stage detectors immediately predict one-step classification and localization. Though they are impractical for deployment on devices with limited processing resources, these models generally outperform the single-stage models in performance. Although these models are not practical for deployment on devices with limited processing resources, they usually perform better than single-stage models. To create resource-efficient object detection models, lightweight object detectors have garnered more attention lately. YOLO series^12–15^ object detectors strike a compromise between speed and accuracy, with the latter reaching real-time performance and enhanced accuracy while operating on GPUs.

Due to the large computational volume, high-performance YOLO models function much less quickly when deployed on edge devices. One of the quickest object identification networks on the market right now is the lightweight Yolov4-tiny, which was suggested as a solution to this problem. Due to its simplistic architecture, real-time performance on low-power devices can be improved, accuracy is constrained, and the extracted features are uniform. Other versions of YOLO are used similarly for other open challenges in object detection.

Additionally, most of the existing YOLO architectures are based on classical activation functions like ReLU or Hard-Swish, which have problems with vanishing gradients and dying neurons, which may affect the performance of the models. Moreover, these models may be manually tuned or tuned through grid search, which does not always give the best results when it comes to hyperparameter tuning. It is evident that models that would provide high accuracy even in complex detection cases, but those with state-of-the-art optimization methods and expressive activation functions are needed. In bridging this gap, in our study, we bring a more perfect and optimal version of the YOLO-NAS architecture, where there is an addition of the MISH activation, which, given that it has a smoother gradient flow pattern and regularization effect, we apply the Enhanced ABC algorithm to automatically search through hyperparameters. With DenseNet-SPAP architecture to obtain robust multi-scale feature extraction, the proposed model is expected to enhance detection rates with relatively small errors, generalization, and real-time speed performance in extreme environments by incorporating these enhancements. This strategy will overcome the shortcomings of the current models directly and offer a more versatile methodology to a variety of real-life circumstances.

In a neural network layer, activation functions are unpredictable point-wise functions that add nonlinearity to the linearly converted input. Selecting the proper activation function is essential to comprehending how well a neural network performs. Mathematically, the application of an activation function in a layer of a neural network is demonstrated by:

1 $$\:x=a\left(y\right)=a\left({\sum\:}_{i}{z}_{i}{j}_{i}+b\right)$$

where x is the output of activation function $\:a\left(y\right)$

Traditionally, sigmoid and Tan functions were used extensively but not in deep learning paradigms. The ReLU activation function is used widely nowadays in deep learning. It has a higher speed of convergence than sigmoid and tanh. ReLU has flaws despite outperforming TanH and Sigmoid in terms of performance and stability. Among these is the so-called Dying ReLU, characterized by an information loss brought on by crushing the negative inputs to zero. Leaky ReLU, ELU, and SELU are just a few activation functions developed over the years to increase performance and overcome ReLU’s limitations. Unlike ReLU, Swish, defined as f(y) = sigmoid (βy), demonstrated significant improvements in outcomes and is a more durable activation function^16^. This work uses the MISH activation function to modify the YOLO-NAS activation function. ReLU and Hard-Swish are the activation functions of YOLO; however, because of their limits, MISH is utilized to improve the model’s performance and detection accuracy.

This research introduces an efficient, finetuned YOLO-NAS framework for real-time object recognition. The proposed model is smaller, reduces the inference time, and achieves better accuracy when compared with YOLOv6, YOLOv7, and YOLOv8. The main contributions of this paper are:

- Since the design of YOLO-NAS uses the commonality of backbone networks (CSPDarkNet or EfficientRep), the baseline of this design is intended to use DenseNet because of its appropriate sharing of features and because it has the advantage of effective context information of multi-scale context, which is covered with Spatial Pyramid Average Pooling (SPAP). This amalgamation provides a considerable enhancement in performance during cases of occlusion, as well as various visual layouts.
- Instead of using traditional ReLU or SiLU activations, the MISH function is employed, which offers smoother gradient flow and self-regularization^16^. This improves model generalization and convergence, particularly useful in deep object detectors with limited data.
- In contrast to YOLO-NAS, i.e., with the generic version of optimizers, Adam or SGD, the framework utilizes the ABC algorithm, during which the hyperparameters of ABC, the learning rate, batch size, and the confidence thresholds, are auto-tuned. Such a new implementation of metaheuristics presents a global optimization element that has never been introduced in the YOLO-NAS pipeline.

This research is organized into different sections. The relevant work of several YOLO series on real-time object detection is presented in “Related survey”. Section “Methodology” provides the introduction of the proposed optimized YOLO-NAS framework. The experimental results and ablation studies are discussed in “Implementation results”. This section also compares the proposed framework with YOLOv6, YOLOv7, and YOLOv8 models. The paper is concluded in “Conclusion”.

## Related survey

Network structure design is one of the prominent issues in deep learning, where many researchers aim to build a network that can be utilized for various tasks, such as detection, segmentation, and recognition. Various techniques have been applied directly to networks like VGGNet^17^, ResNet^18^, and GoogLeNet^19^, yielding satisfactory results. To create a more meaningful feature map, SSD^20^ uses VGG16^21^ as the backbone. Similarly, ResNet utilizes VGG-16 for medical diagnosis. Therefore, in this study, DenseNet is utilized with YOLO-NAS.

Recent approaches to object detection have increasingly tried to move to Transformer-based designs mainly because of the success generated with DETR (DEtection TRansformer) and its derivatives^22^. This work proposes a new solution to the problem of object detection based on replacing the classic region proposal network architecture with a Transformer encoder-decoder model and, as a result, performing end-to-end object detection without making any passes through non-maximum suppression and anchor boxes. In addition, the T-DETR optimizes feature encoding and cross-attention to get the model running in real-time with reduced convergence time in comparison to DETR. Although these models can attain high rates of accuracy, they consume a lot more computational resources than CNN-based models such as YOLO. In addition, their rate of inference on restricted devices is an issue, and these lightweight and efficient versions, like YOLO-NAS, would be useful in creating real-time applications under edge deployments.

Most deep neural network-based object detection algorithms were two-stage techniques before YOLO. YOLO deftly solves the detection challenge using regression, a one-stage approach. This method preserves good detection results while dramatically increasing detection speed. As a result, users across various industries have developed and made the YOLO algorithms increasingly famous. Table 1 provides a brief related work with future scope or research gaps in the YOLO series.

**Table 1:** Brief related work with research gaps and future scope in the YOLO series.

Author	Dataset	Model	Results	Research Gap
Alina Ciocarlan et al.^23^	NUAA-SIRST, IRSTD-1 K datasets	A deep contrario framework consisting of an NFA module is proposed.	The model achieved better results when compared with other SOTA models for detecting tiny objects.	The model is not able to produce bounding boxes for real-time object detection.
Sri Padma et al.^24^	Kaggle image dataset and live stream videos are used to capture the images	YOLOv2	The paper presents a comprehensive study of YOLOv2 and improved YOLOv2 for face mask detection. The improved model achieved an accuracy of 95%.	The profounded model cannot detect the face mask of persons more significant than two in any image.
Sijie et al.^25^	PASCAL VOC07 + 12, MS COCO datasets	A lightweight YOLOv4 detection model is suggested, with MobileNetV2-CA as the attention mechanism in security contexts instead of the backbone model.	The model achieved mAP of 74.73%.	In the future, accuracy loss and detection accuracy will be enhanced. The model will be optimized to attain better detection accuracy.
Zhengwei et al.^26^	Drone vehicle dataset and UCAS-AOD dataset	A lightweight rotational YOLOv5 (R-YOLOv5) is proposed for vehicle detection in dense scenes.	The model’s accuracy for both datasets was 84% and 90%, respectively.	The model cannot predict the images of very small objects occluded and illuminating light conditions.
Chhaya Gupta et al.^27^	MS-COCO dataset	A fine-tuned transfer learned YOLOv6 is introduced for real-time object recognition.	It produced better results by comparing the suggested model to SSD, FasterRCNN, Mask RCNN, YOLOv4, and YOLOv6.	A kernel pruning algorithm will be applied to improve the detection accuracy further.
Ignazio et al.^28^	UAV dataset in agriculture	YOLOv7 is utilized to detect crop weeds in the Chicory plant.	The model achieved a mAP of 56%.	The model is not improved enough to detect crop weeds of all types.
Abdur et al.^29^	MS-COCO dataset	YOLOv7 is used to detect and count vehicles in real-time.	The model produced favorable outcomes.	The model was not able to detect fast-moving vehicles in videos.
Armstrong et al.^30^	Custom dataset	YOLOv8 is used to detect helmet violations in real-time video frames.	With a mAP of 58.61%, the model produced satisfactory results.	The model can be enhanced further with transfer learning algorithms for better detection accuracy.
Haitong et al.^31^	Tiny person dataset, PASCAL VOC 2007 dataset	Using camera sensors, DC-YOLOv8 is suggested for real-time object detection.	The model performed well compared to YOLOX, YOLOR, YOLOv3, scaled YOLOv5, YOLOv7-tiny, and YOLOv8.	The model cannot detect a person sitting in a fast-moving vehicle without a helmet.
Yugen Yi et al.^32^	DUTS, HKU-IS, PASCAL-S, ECSSD, DUT-OMRON datasets	A GPONet optimization network merged with a gate fusion network is proposed for salient object detection.	The model achieved good results when compared with other SOTA models.	The model detected some non-salient objects, and segmentation was improper.
Arpita Dutta et al.^33^	eBDtheque, DCM, Manga, BCBId, COMICS datasets	For comic emotion analysis, a framework named EmoComicNet is proposed.	The model achieved better results.	The model is limited to Bangle and English comic datasets only.
Priyanka et al.^34^	YOLO-NAS	Tropical cyclone intensity is estimated using the YOLO-NAS model with the help of satellite images in real-time.	The model achieved an accuracy of 81%.	The model depends on image quality and computational and real-time processing constraints.
Nguyen et al.^35^	YOLO-NAS	Container damage detection using deep learning model.	The model achieved a mAP of 91.2%.	Despite its advantages, the model faces limitations, including its reliance on high-quality, annotated datasets and potential challenges in detecting damage in cluttered or occluded environments. Minor defects, such as rust stains, may also go undetected, and its high computational demands can pose difficulties for resource-constrained settings.
Anil Kumar et al.^36^	RMFD, IMFD, and real-time video captures	MobileNetV2 with transfer learning (CAFFE)	Achieved 95.3% detection accuracy for masked/unmasked faces; inference tested on webcam	No support for multi-object environments; lacks real-time speed benchmarking (FPS); no metaheuristic tuning included.
Anil Kumar et al.^37^	Custom masked face dataset	Modified MobileNetV2, CAFFE framework, Transfer Learning	Achieved 91.2% accuracy for masked face age prediction, with robust performance in low-light and real-time conditions.	No cross-dataset validation; lacks integration with object detection pipelines like YOLO; no optimization strategy applied.

From the vast literature review, it can be concluded that most of the YOLO versions cannot detect occluded objects, illuminated objects in light conditions, and small objects. Hence, to solve the problem, a finetuned and optimized YOLO-NAS model is proposed.

## Methodology

This section comprises the executed methods and the dataset used for the study. An explanation of the dataset used for model testing and training is also provided.

### Dataset and experimental setup

#### Dataset

The work uses the MS-COCO dataset for training the fine-tuned and optimized YOLO-NAS model^38–40^. The MS-COCO dataset is used to train the models, and a custom dataset is also collected for testing purposes. The training images are brightened and positioned more precisely to avoid overfitting and provide an efficient object detector. The dataset has a total of 123,287 images with different classes. The dataset is split into an 80:20 ratio, with 80% of the photos being used to train the models and the remaining 20% being used to test the models. The primary scope of this research is to focus on persons, cars, traffic lights, cats, dogs, benches, umbrellas, and bus classes. The size of an image is 416 × 416. For testing the models, random images and videos are collected by the authors to evaluate the performance of all the models.

To test the efficiency of the proposed object detection framework based on YOLO-NAS under the conditions of the real world, the so-called custom dataset was created with the help of a combination of the YouTube videos frames and the pictures sourced by curators. When this dataset is interpreted, the goal is to simulate the randomness and inconsistency found in real deployment scenarios like those found in a roadside environment, people monitoring, road conditions, and mobile hand-held tracking. The dataset will include 200 high-resolution pictures extracted from various community bytes available on YouTube and other community-added content. The selection of the videos was also based on the abundance of the types of objects (e.g., vehicles, pedestrians, road signs), complexity of the scene, the diversity of light conditions, occlusion, and motion blur. With a 1 FPS setting, there are limitations to how redundant frames are extracted as the process prefers diversity. The process of labeling was completed using the PASCAL VOC format, with severe attention being paid to the accuracy of the bounding boxes using LabelImg.

#### Dataset splitting strategy

To achieve no leak of data and to ensure that everyone would have equal chances of evaluation, the data was divided into three subsets before transformation or optimization: Ratio of training was set as 0.7 out of all the data The coverage is 15 per cent of data The total number is 15 for the sample that is going to be tested. The balance between classes has been carried out through stratified sampling to ensure that each subgroup has a balance between classes. Notably, it means that the test set was completely separated in the process of design, training, and tuning of the model, as well as in the process of optimization of the features to be used.

#### Data preprocessing

The size of all the images was adjusted to 640 × 640 to agree with the model input size. Another step was to scale the pixels by their mean and standard deviation as set out in the standard ImageNet statistics. Randomly flipped, adjusted in brightness, scaled, and rotated to increase robustness, but used after being applied to the training set. The validation or test sets did not involve enhancement or distortions of any kind except resizing and normalizing.

### Models used for the study

This section describes all the models used in this study for evaluation purposes.

#### YOLOv6

**Fig. 1:**
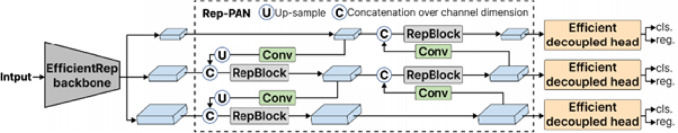
The architecture of YOLOv6^42^.

“You Only Look Once version 6,” or “YOLOv6,” is an object identification system that compromises speed and accuracy by utilizing state-of-the-art methods. It’s also known as MT-YOLOv6 or Meituan-YOLOv6 because a group of developers from Meituan, a Chinese e-commerce firm, developed it^41^. This model, which consists of the Head, Neck, and Backbone, is true to the progenitors’ threefold anatomy, as shown in Fig. 1. To further enhance its distinctiveness in the field, YOLOv6 sets itself apart by presenting an anchor-free model with a reparameterized backbone. This figure abstracts the fact that an advanced object recognition model has been constructed based on an EfficientRep backbone and Rep-PAN neck, with efficient decoupled detection heads. The OptimRep backbone is the first stage that gets the initial representation of the input image as a hierarchical representation of features. The resulting feature maps go into the Rep-PAN module, which is supposed to further improve multi-scale feature fusion. The feature maps are a good process carried out in RepBlock units and in convolutional maps in Rep-PAN. There are up-sampling operators in every path that have been incorporated to ensure that there is spatial resolution within the path, and the concatenation of the channels has been introduced to ensure that there is merging of the information across the scales. The combination of the features enables the ensemble to grasp the fine-grained and semantic individuality. The branching of the three different resolution outputs then feeds to separate efficient, albeit unbound, decoupled heads, and where each of them, independently, predicts through classification scores (cls.) and bounding box regression (reg.) outputs. The decoupling allows the network to learn classification and localization separately from each other, and this improves accuracy and avoids instability in the convergence. Altogether, this architecture is a very trade-off between computation and high detection performance.

The YOLOv6 architecture’s backbone is its central component and is vital in feature extraction. Most of the processing power is carried by the neck and head regions of the network, which get input from the extracted features. Repameterized backbones are provided by YOLOv6, which addresses the competing needs for speed and accuracy that are frequently present in linear networks like VGG and classic multi-branch networks like ResNets^27^. Like other object detection models, the neck of YOLOv6 uses Path Aggregation Networks (PAN) to harvest multi-scale feature maps. RepPAN in YOLOv6 is a novel design combining functionality from several reparameterized blocks, increasing hardware-friendliness.

A notable change from YOLOv5 is the introduction of the Efficient Decoupled Head. Because of this unique architecture, the identification and classification branches efficiently cut processing requirements while increasing accuracy because they no longer exchange parameters and branch off separately from the backbone. Finally, YOLOv6 employs two distinct loss functions: Distributed Focal Loss (DFL) is used in conjunction with an SIoU or GIoU for box regression, and Varifocal Loss (VFL) is used for categorization. By giving positive and negative examples with different weights and levels of relevance during training, VFL, a form of focus loss, manages both basic and complex scenarios with efficacy. Adequate learning signals from both kinds of data are encouraged by this method. The box regression loss in medium and large YOLOv6 models is calculated using DFL, which treats the box locations’ continuous variance as a segmented probability distribution. This method works exceptionally well in situations when the ground truth bounds are not clearly defined^27^. This work proposes a pruning algorithm to help fine-tune and prune the YOLOv6 model in^27^ by the authors.

#### YOLOv7

**Fig. 2:**
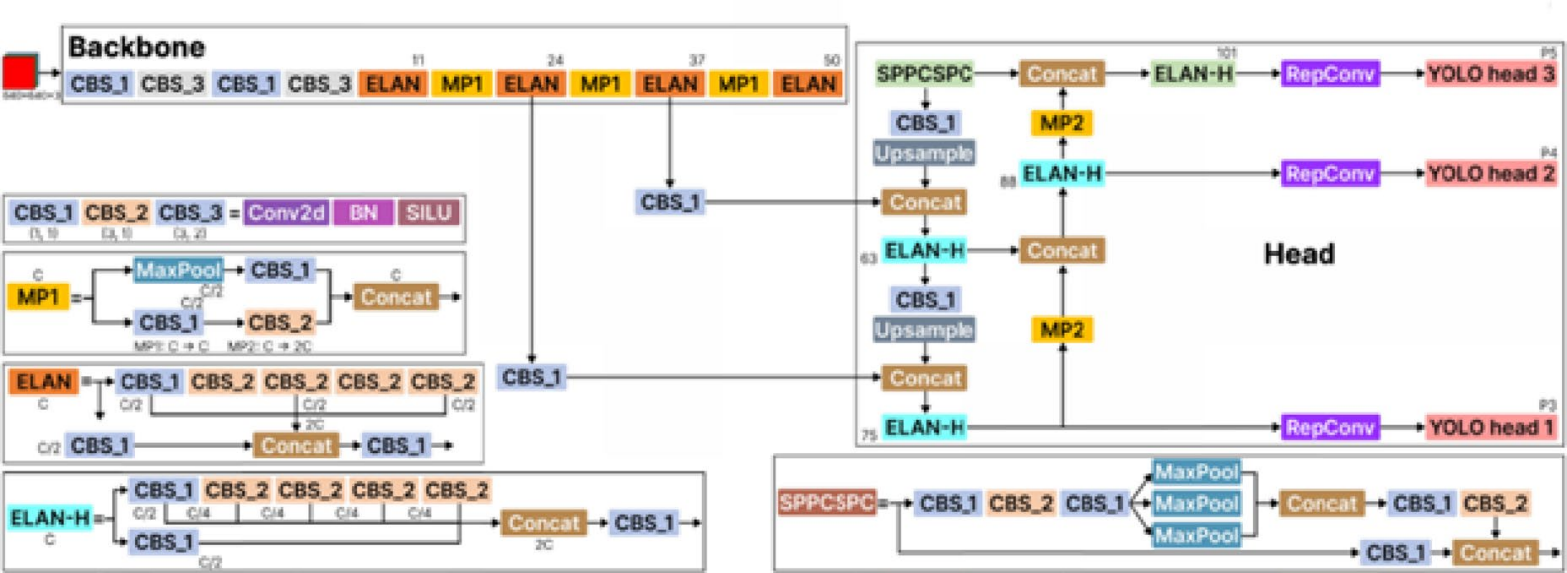
The architecture of YOLOv7^42^.

“You Only Look Once Version 7”, often known as YOLOv7, represents a notable breakthrough in object detection algorithms. It is built on the widely recognized head, neck, and backbone (YOLO) framework, as seen in Fig. 2. Each element is essential to object detection and image recognition. The goal of YOLOv7’s development was to create a network architecture that could, at the same inference speed, predict bounding boxes more accurately than other models of a similar type. This was accomplished by making several significant changes to the YOLO network and its instruction protocols, which enhanced the functionality of each YOLO framework component. YOLOv7 has several improvements, including the Extended Efficient Layer Aggregation Network (E-ELAN), a larger variant of the ELAN computational block. Because of this modification, the convolutional layers at the backbone of the YOLO networks function more effectively, which is essential for maintaining good inference speed. It takes into account the amount of memory needed for layer retention as well as the distance the gradient must travel backward through the layers; a shorter gradient is preferred to improve the network’s learning abilities^43^.

Re-parameterization planning is a novel addition to the network. This procedure specifies the average of model weights to create a more reliable model for the patterns. The network modules that ought to implement these tactics were identified using gradient flow propagation pathways. Furthermore, Yolov7 uses model scaling strategies to recognize that different applications call for differing precision and inference rates. By concatenating layers and scaling the network’s width and depth simultaneously, this method helps preserve the best possible model architecture over various sizes. Additionally, the Supplementary Head Coarse-to-Fine idea is presented in YOLOv7. Along with the head that effectively generates the predictions, an auxiliary head in the system’s center is observed during training. Different levels of supervision were tested for the auxiliary head, and because of its close resemblance to the prediction, it does not train the final head. YOLOv7’s novel features and improvements represented essential advancements in computer vision research. With improved speed and accuracy, model scaling, modifications to the architecture, and a new coarse-to-fine technique, YOLOv7 stood out as a significant advancement in object recognition capabilities. In this paper, the YOLOv7 model is finetuned with the help of the proposed algorithm in^27^.

#### YOLOv8

**Fig. 3:**
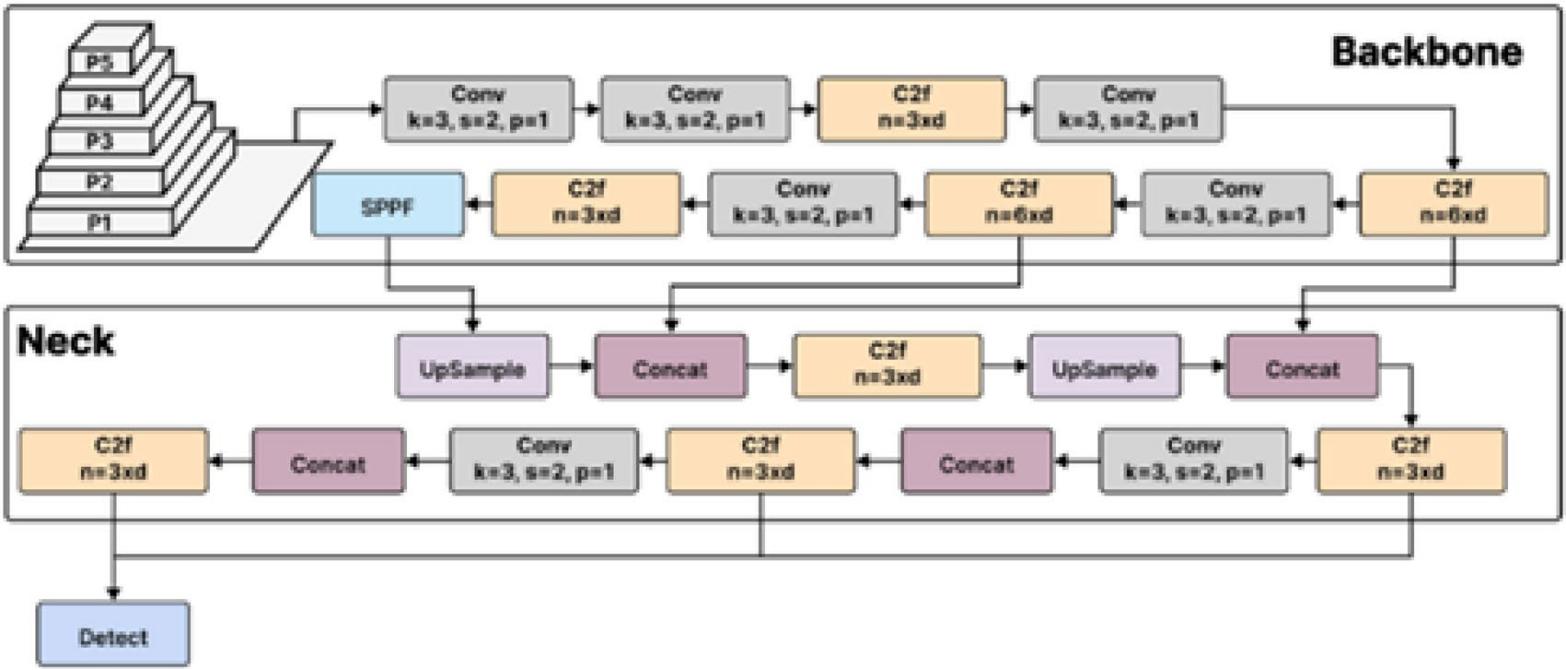
The architecture of YOLOv8^42^.

Ultralytics unveiled YOLOv8, the newest model in the popular “You Only Look Once” line, on January 10, 2023. Building on the foundation created by the popular YOLOv5 model, YOLOv8 represents a significant breakthrough in instance segmentation, object identification, and image classification. Although there isn’t a published paper yet, the community conversations and the repository offer valuable perspectives into the revolutionary changes brought about by YOLOv8. YOLOv8 follows the three primary architectural elements—the Backbone, Neck, and Head—that characterize the YOLO framework, much like its predecessors did. These components work together, as illustrated in Fig. 3, with the Head predicting item locations and classes, the Neck integrating these characteristics, and the Backbone supplying characteristics to the image frames. Instead of the anchor-box technique utilized in previous YOLO models, an anchor-free model is one of YOLOv8’s most notable characteristics. With this innovative modification, the model can now directly estimate the center of an item, mitigating the limitations of anchor boxes, such as their poor generalization and incapacity to deal with anomalies. YOLOv8 speeds up the Non-Maximum Suppression (NMS) procedure, a critical post-processing step in charge of sorting through candidate detections following inference, by lowering the number of box predictions.

Additionally, YOLOv8 introduces architectural improvements in convolutions, the essential neural network building blocks. Adding C2f, which takes the place of C3, and substituting a 3 × 3 convolution for the original 6 × 6 convolution in the stem have made the model structure more effective and adaptable. The new design concatenates all of the outputs from the Bottleneck, in contrast to the C3 arrangement, which only used the output of the previous Bottleneck. It is composed of two residually connected 3 × 3 convolutions. This improvement maximizes YOLOv8’s potential in computer vision applications by guaranteeing a more robust model structure. As a noteworthy development in the Yolo series, YOLOv8 raises the bar even higher for computer vision projects in the future. With its high accuracy rates, creative architectural modifications, and better developer options, YOLOv8 keeps improving and is becoming a more and more desirable option for real-time object identification. YOLOv8 is used for underwater trash detection in^44^ which is finetuned with the help of the pruning algorithm proposed in^27^. This work uses a proposed pruning technique to optimize the YOLOv8 model^27^.

#### YOLO-NAS

**Fig. 4:**
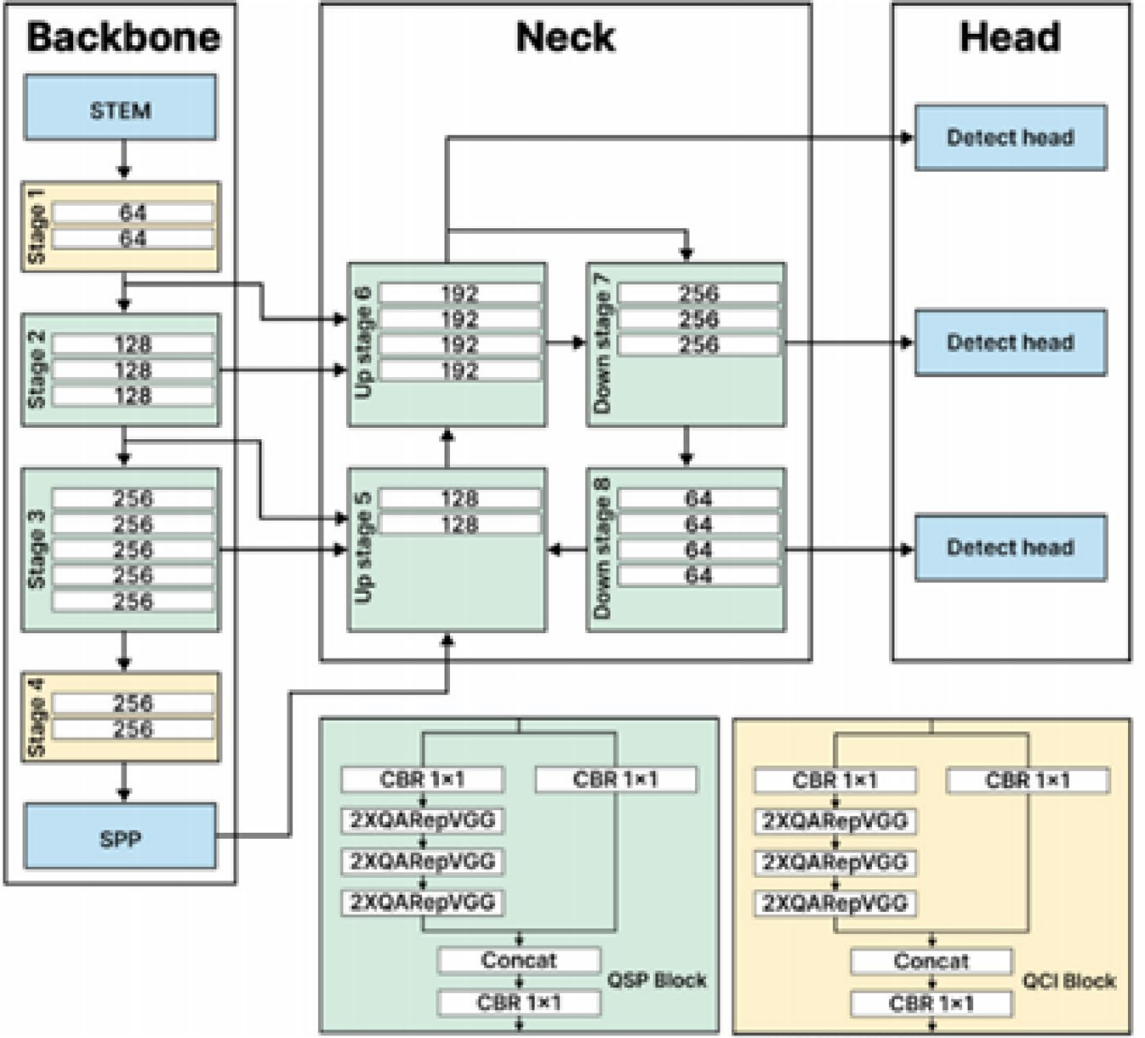
The architecture of YOLO-NAS^42^.

YOLO-NAS is a basic novel object detection model. It results from carefully considered Neural Architecture Search technology, which was developed to overcome the shortcomings of earlier YOLO models. YOLO-NAS is a significant advancement in object identification with notable gains in accuracy-latency, trade-offs, and quantization support. To achieve the best performance, YOLO-NAS utilizes selective quantization and quantization-aware blocks. The model performs far better than other YOLO models when converted to its INT8 quantized version, with only a tiny precision decrease. These improvements add to an exceptional architecture that performs well and can recognize objects. Figure 4 shows the architecture of the model.

One area of specialization within AutoML (automatic machine learning) is NAS (Network Architecture Search). Deep neural network architectures are created using machine learning models to surpass their manual counterparts developed, configured, and constructed by human researchers. The development of NAS research makes it easier to find high-performing neural networks by using a systematic approach that considers variables like prediction accuracy, inference speed, and computing resource availability. Regarding tasks like object identification, image classification, and natural language processing, the successful efforts of innovative architectural design and development made possible by NAS approaches have outperformed human-engineered systems. A critical advantage of applying NAS approaches is that it can reduce the number of hours that researchers must spend on human labor by streamlining machine learning procedures through automation. Furthermore, scarcity or a lack of expert domain knowledge is addressed by introducing systems that can explore various internal network configurations in an ample search space using brute force and considering multiple parameters to optimize. This process eventually establishes the optimal network with configurations that, in certain situations, can only be accomplished with human expert knowledge, creativity, and intuition. Because they concentrate on optimizing elements like computing resources, efficiency, accuracy, power consumption, and memory usage—all essential for modifying architectures to fit edge devices (smartphones) or real-time scenarios—NAS techniques are even more pertinent nowadays.

A critical aspect of YOLO-NAS is incorporating Quantization-Aware RepVGG (QA-RepVGG) components within the model’s architecture. These building pieces minimize accuracy loss during Post-Training Quantization (PTQ) by guaranteeing compatibility. The model supports 8-bit quantization and reparameterization with its unique “QSP” and “QCI” modules comprising QARepVGG blocks. The creation of YOLO-NAS represents an essential advancement in object detection, albeit without an official publication. Its exceptional performance in inference latency and mAP metrics suggests it could be helpful to for real-time, high-demand detection applications. It is believed that this unique model’s entire strength and potential will become apparent when more details about its training program become accessible. YOLO-NAS represents the state-of-the-art in real-time object detection and gives the YOLO series a new angle with its sophisticated automated architecture design methodology.

#### Pruning the models

Using a feature score as a guide, pruning reduces the redundancy of a network. As a result, less computation is required to generate a lower-dimensional network than the baseline network. The three steps of pruning are fine-tuning pruning, sparsity learning. Sparsity learning networks are the primary foundation for pruning. Unwanted parameters are identified during trimming and eliminated based on their feature ratings. By using this method, the number of parameters in any neural network is reduced to reduce the dimensionality. Retraining the network requires fine-tuning the remaining parameters after removing the undesirable ones.

Pruning is reducing the model’s weights to still function well in difficult situations. It reduces the model’s scale and hits a network’s layer structure. Pruning is mainly used to remove filters that are deemed unnecessary while assessing the network’s overall performance. Before removing the framework’s layer structure, each layer’s performance is assessed, and the layer with the least impact on the model’s recognition accuracy is selected and trimmed. Iteratively, this is carried out until the extra validation loss beyond the “post-pruned threshold.” The post-pruned threshold is the ratio of the pruned model’s validation loss to the entire model’s. Next, the model undergoes a retraining process to reduce the validation loss below a level referred to as the “post-retrained.” The post-retrained threshold is a percentage of retrieved validation loss compared to the entire model’s validation loss. After successfully retraining the model, the process terminates when the validation loss of the pruned model surpasses the post-pruned threshold. This algorithm is known as single-layer pruning since it only prunes one layer at a time. After one of the model’s layers is changed, the validation loss is computed to identify which layer should be pruned next. When a layer is modified, undesirable features are eliminated. All the features’ sum absolute weights (SAW) are computed to determine undesirable features. Pruning occurs for the characteristics having the lowest SAW.

This research employs a hidden layer pruning technique to make YOLO-NAS a lightweight network by lowering the number of features and the network depth. Model trimming results in a drop in detection accuracy. A transfer learning technique was applied to overcome this situation and improve detection accuracy. In this paper, the entire algorithm is thoroughly detailed. The algorithm is summarized as follows:

**Figure:**
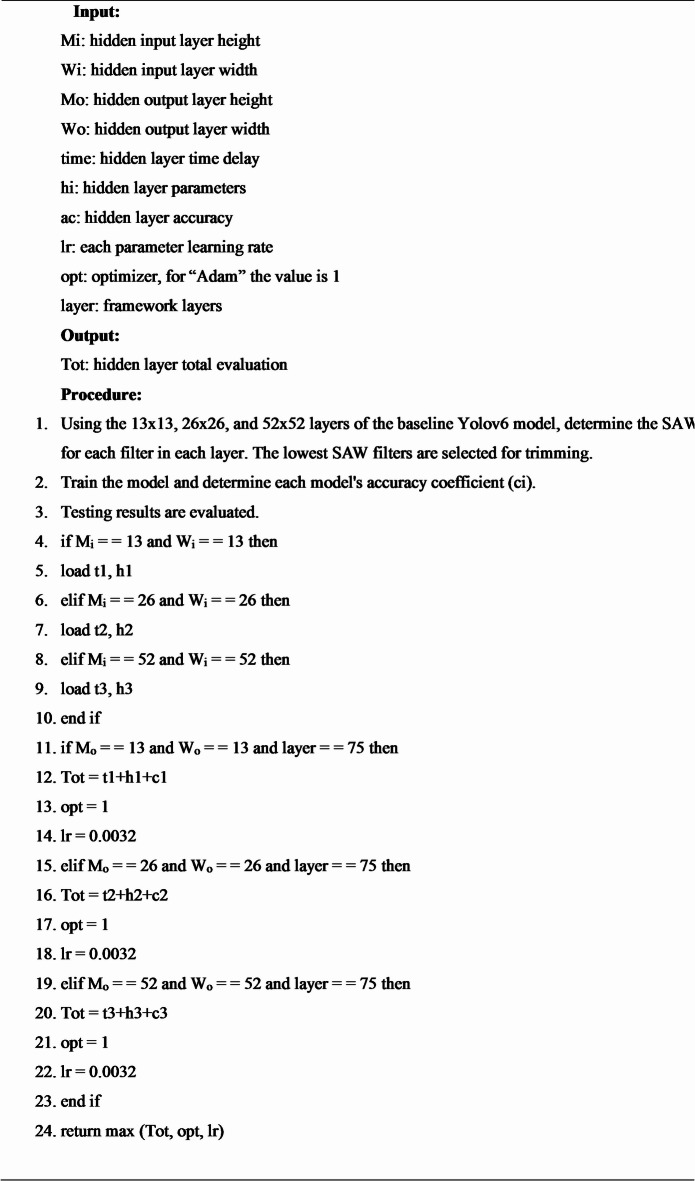
**Algorithm 1** Pruning algorithm^27^.

#### Optimization of the model with the ABC method

Hyperparameters are essential for optimizing deep learning algorithm training. Carefully choosing the model’s hyperparameters can significantly improve its performance once the appropriate model and dataset have been chosen. Making accurate adjustments to the hyperparameters can significantly improve the model’s performance. The hyperparameters of the YOLO-NAS algorithm are divided into two main groups: those connected to data augmentation techniques, such as scale, hsv, and mosaic, and those fundamental, such as learning rate, loss function, and momentum.

The artificial bee colony optimization technique was introduced by Dervis et al.^45^ in 2005. How honeybees forage in their habitat, where food sources are viewed as potential treatments and the most effective food source, is a good signal of the most significant medicine and inspired the ABC algorithm. The ABC algorithm is frequently used in various industries and applications due to its simplicity and flexibility.

The bees in the ABC algorithm are divided into three groups: employed bees, observation bees, and scout bees, depending on the tasks they are assigned. While employed bees oversee keeping their plentiful resources, scout bees are haphazardly searching for food supplies in new areas, much like a global search. Observer bees rely on information from working bees in their search for more reliable sources. The ABC algorithm randomly selects the starting location of food sources and establishes the initial values for the control parameters. This yields the starting population, which is expressed mathematically in Eq. 2 based on the lower and upper bounds of the variables that have been determined.

2 $$\:{y}_{ab}={y}_{b}^{min}+rand\left(\text{0,1}\right)({y}_{b}^{max}-{y}_{a}^{min})$$

where$$\:{y}_{b}^{max}=upper\:bound,\:{y}_{b}^{min}=lower\:bound,\:{y}_{ab}=sol.\:vector,\:a=\text{1,2},\dots\:N$$; $$b=\text{1,2},\dots\:.,NS;N=no.\:of\:parameters\:and\:NS=no.\:of\:solutions.$$

The algorithm describes the ABC algorithm. The algorithm is based on a counter mechanism that works as per the greedy solution. The counter works, for example, if the proposed algorithm is better. Otherwise, the counter value is increased. All the models used in this experimental study are optimized with the proposed ABC algorithm and then executed for evaluation.

The probability-based selection approach used by the ABC algorithm is represented by Proi, which stands for the likelihood of selecting a solution, and Qi, which stands for the solution’s quality. Here, a greedy screening mechanism is used, like the previous bee phase. The experimental number for this solution is raised if the fresh solutions are not superior to the current ones. When a food source in the ABC algorithm can no longer be made better, it is exhausted. Whenever a solution is abandoned, it is determined by the control parameter “l”, which stands for limit. When a solution receives more trials than permitted, the source is deemed useless, and the bee associated with the source is removed. In this case, the solution is called a “scout bee.”

**Figure:**
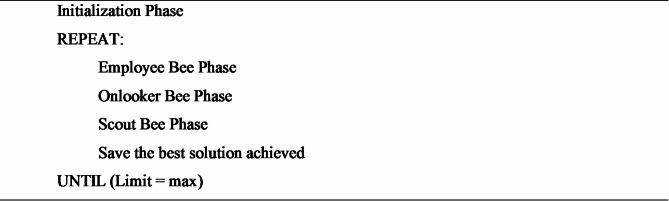
**Algorithm 2** Artificial bee colony algorithm.

The initialization phase is defined by Eq. (2) stated above. The scout bees initialized the population of food sources that are defined by the vector $\:\stackrel{⃑}{{y}_{ab}}$. Each food source vector is a solution to the optimization problem.

During the employee bee phase, the bees search for new food sources that are more nectar-rich and closer to their memory of the food source. They find a food supply close by and evaluate its quality or viability. This phase is defined as:

3 $$\:{\vartheta\:}_{ab}={y}_{ab}+{{\varnothing}}_{ab}({y}_{ab}-{y}_{kb})$$

where $\:{\vartheta\:}_{ab}$ is the new food source, $\:{y}_{kb}$ is the randomly selected food source, and $\:{{\varnothing}}_{ab}$ is a random number.

The quality of the solution ($\:{Q}_{ab}\left(\stackrel{⃑}{{y}_{ab}}\right))\:$is calculated as follows:

4 $$\:{Q}_{ab}(\stackrel{⃑}{{y}_{ab})}=\left\{\begin{array}{c}\frac{1}{1+{ob}_{ab}(\stackrel{⃑}{{y}_{ab})}}\:\:\:if\:{ob}_{ab}\left(\stackrel{⃑}{{y}_{ab}}\right)\ge\:0\\\:1+abs({ob}_{ab}\left(\stackrel{⃑}{{y}_{ab}}\right)\:\:if\:{ob}_{ab}\left(\stackrel{⃑}{{y}_{ab}}\right)<0\end{array}\right.\:$$

where $\:{ob}_{ab}$ is an objective function that gives the value of $\:\left(\stackrel{⃑}{{y}_{ab}}\right)$ solution vector.

The onlooker bee phase has two groups of bees: onlookers and scouts. The observer bees remain within the hive until the employee bees advise them of a food supply. After receiving the information from the worker bees, the spectator bees choose their food sources using a probabilistic approach and the quality values of the offered solutions, as indicated:

5 $$\:{Pro}_{ab}=\frac{{Q}_{ab}\left(\stackrel{⃑}{{y}_{ab}}\right)}{\sum\:_{ab=1}^{NS}{Q}_{ab}\left(\stackrel{⃑}{{y}_{ab}}\right)}$$

The unemployed bees who choose their food randomly are known as scout bees. When an employee bee’s solution can no longer be improved even after several attempts, they become scout bees, and their methods are abandoned. At random, these scout bees begin to discover new solutions. After all the phases of the ABC algorithm have been achieved, the best solution is saved for further use.

The Enhanced artificial bee colony (EABC) Optimization technique is an improved version with a swarm intelligence-based optimization method inspired by the foraging behavior of honey bees. Standard ABC sometimes suffers from slow convergence due to an imbalance between exploration (global search) and exploitation (local search). The enhanced version modifies the solution update equations to enhance these two aspects’ trade-offs. The way employed bees generate new solutions is modified to reduce premature convergence. Onlooker bees use an improved selection mechanism to choose high-quality food sources more efficiently.

The EABC optimization technique can significantly improve the performance of YOLO-NAS in real-time object detection by optimizing various aspects of the model. It fine-tunes YOLO-NAS’s hyperparameters for better accuracy and speed, such as confidence threshold, learning rate, batch size, and weight decay to remove the issues of underfitting and overfitting, as well as anchor box size. The modified values of these hyperparameters are shown in Table 2. This enhanced version of the ABC optimization technique optimizes the neural architecture of YOLO-NAS by selecting the best layer configuration and reducing unnecessary computations. It also helps prune redundant parameters while maintaining accuracy and enhances frames per second in real-time detection. NAS uses evolutionary algorithms, and the enhanced ABC optimization technique further refines the model structure by improving layer connectivity and depth. This technique optimizes the feature extraction layer in YOLO-NAS by selecting the most relevant features for detection.

An improved ABC algorithm was used to optimize the YOLO-NAS model with a particular emphasis placed on the process of tuning the most significant hyperparameter values and activation parameters. The optimization setup used in this study was the following:

The fitness metric adopted in ABC was the average precision (AP) that was calculated by considering 3 IoU thresholds, namely 0.50, 0.75, and 0.95. The fitness metric is defined as:

6 $$\:fit=\frac{mAP@0.50+mAP@0.75+mAP@0.95}{3}$$

It is this composite scoring that sees that the solutions chosen are not only great in terms of their simple detection accuracy but also can enhance their performance under tighter IoU constraints, optimizing their overall localization and generalization.

All the choices in the ABC algorithm are architectural options and sets of hyperparameters that can be optimized and utilized within the context of YOLO-NAS. The size of the population in the ABC population was sampled to 70 due to a set of preliminary tests to balance exploration with usable computation. Each ABC run was executed for 100 iterations (cycles), ensuring adequate convergence behavior while avoiding overfitting to local optima. Termination was also conditioned on stagnation: if no fitness improvement was observed in 20 consecutive iterations, the run terminated early. To ensure the robustness and to reduce the variance, the optimization algorithm of ABC was performed 10 times, and at each iteration, the optimization procedure was started with a different random seed. The single best solution (regarding average mAP score) performed at all runs was to be used to do the final model evaluation.

**Table 2:** Values of hyperparameters optimized with the enhanced ABC optimization technique.

Hyperparameter	Value
Confidence Threshold	0.5
Learning rate	0.003
Batch size	32
Weight decay for regularization	5e-4
Activation function	MISH
Anchor box optimization	Based on the size of the dataset

To determine how much the ABC optimization process can be consistent and effective, 10 separate optimization runs were executed with various random seeds and the results are shown in Table 3. The goal of every run was to maximize the mean mAP at the IoU thresholds 0.50, 0.75, and 0.95, as the sum of the terms of the fitness function, and the results are shown in Table 3. The outcome demonstrates that the ABC algorithm was sufficiently certain of good-performing hyperparameter settings, and the standard deviation of the runs does not indicate large differences (SD = 0.83%). The top-performing setup laid down a composite MAP of 92.56%, whereas the worst-performing setup still laid down 89.72%, which is a healthy indication of the strength of the optimizer exploring the non-convex search space.

**Table 3:** Statistical result of fitness function after 10 runs.

Metric	Value (%)
Best fitness (mAP)	92.56
Worst fitness (mAP)	89.72
Average fitness (mAP)	91.34
Standard deviation	$\:\pm\:$ 0.83

**Fig. 5:**
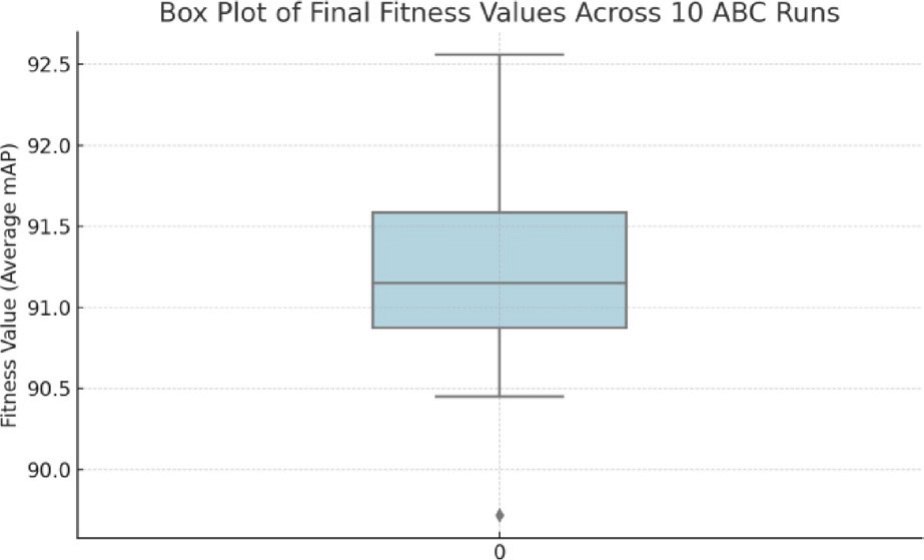
Box plot showing the distribution of final fitness values (average mAP) across 10 independent runs of the ABC algorithm. The model demonstrates high stability with low variance and no significant outliers.

**Fig. 6:**
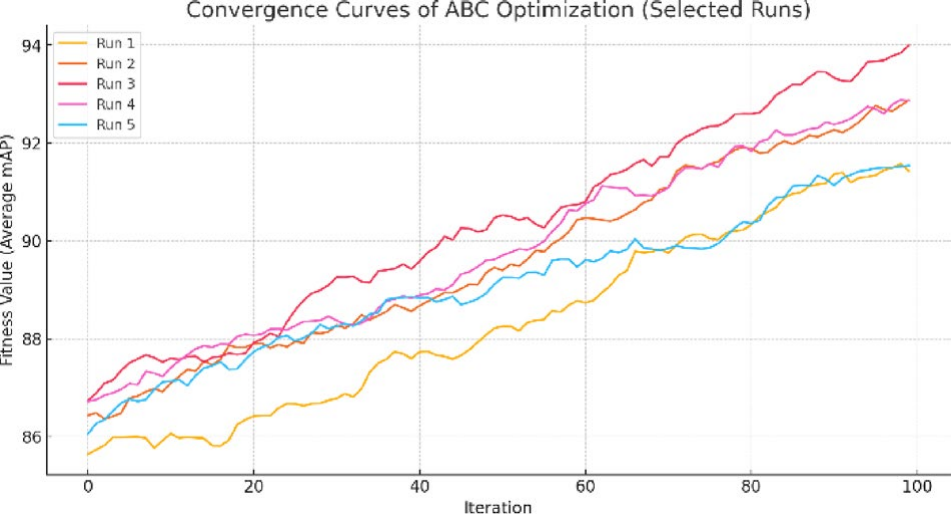
Convergence curves of the ABC algorithm across selected runs. The optimizer shows rapid fitness improvement in the early iterations, followed by steady convergence, demonstrating both strong exploration and exploitation phases.

To empirically understand more about the behavior of the ABC optimization process, we did two things, one of which is plotting of the final fitness distribution of the run at 10 independent runs as shown in Fig. 5, and the other is the convergence plot of the iterations in Fig. 6. The box plot could facilitate the comprehension of the fact that the optimizer gives rather good, regarding very low variance and the lack of outliers, solutions on a regular basis. In the meantime, the convergence curves underline the presence of rapid early-stage gains followed by stabilization, which are the signs of a healthy ratio between worldwide exploration and domestic exploitation. These artifacts confirm the trustworthiness and the soundness of ABC to streamline the YOLO-NAS model.

The ABC optimization algorithm was chosen in this study because of its simplicity, high global search abilities, and the successful application of the optimization algorithm in other fields in solving various complex problems, with the latest of which being the training of neural networks and the tuning of neural network parameters. Based on the behavior of the honeybee swarms (foraging), ABC incorporates the parameters of the exploration (global search) and exploitation (local refinement), which is necessary in the high-dimensional and non-convex search space, deep learning models, such as in the case of YOLO-NAS. Among the metaheuristic algorithms, other algorithms such as the Reptile Search Algorithm (RSA), Red Fox Optimizer (RFO), are relatively new and their performance has been catching up with those of the older algorithms, but during the selection of an optimization algorithm, there is need to consider not only the performance measure but also the simplicity of the algorithm and how easy it can be integrated with ease of interpretation and the robustness of such algorithm as determined empirically. An upside of ABC, in this respect, is a reasonable trade-off between computational cost and the accuracy of optimization. The behavior-driven search approach that characterizes its search algorithm is less prone to early convergence as compared to some of the population-based algorithms, and it is especially suited to real-time applications where ease of implementation is paramount.

The implications of the No Free Lunch (NFL) Theorem of Wolpert and Macready, who argue that there is no optimization algorithm that surpasses all others in terms of searches over all possible problem sets, are also known. Such a fundamental principle upholds the argument that the success of an optimization algorithm is always problem-specific. Therefore, even though RSA and RFO can potentially be more successful than ABC in restricted optimization or benchmarking of functions, ABC was effective in this experiment due to the real setting of the objective landscape of hyperparameter optimization of YOLO-NAS under consideration.

In addition, the modularity of ABC enabled the proposal of a modularity-enhanced variant to incorporate a quality-based pruning scheme and a performance-sensitive selection policy, based on the mAP metric that best fits the distinct demands of real-time object detection. A comparative testing of ABC, RSA, RFO, and similar nature-inspired optimizers could also be done in the future to increase their validation and optimization of the framework to allow further deployment.

#### Motivation for ABC optimization

The ABC algorithm is used as a meta-heuristic search algorithm of elite hyperparameters, which can be employed to optimize the performance of the YOLO-NAS framework. ABC is most suited to global hyperparameter tuning, whereas gradient-based optimizers, like Adam or SGD, are suitable to train the weights of neural networks; the latter have no issues when the search space in question is high-dimensional and non-convex. The parameters that are therefore important to tune are: learning rate, confidence level, batch size, and weight decay. The choice of ABC is based upon its population-based search strategy, through which it becomes possible to be robust in search and exploitation. Furthermore, a custom fitness was provided with a weighted sum of mAP and the latency of inference, so that it was also feasible to use the model in real-time. This makes ABC an expedient and reactive possibility in applications where compromises must be made regarding speed and accuracy.

**Fig. 7:**
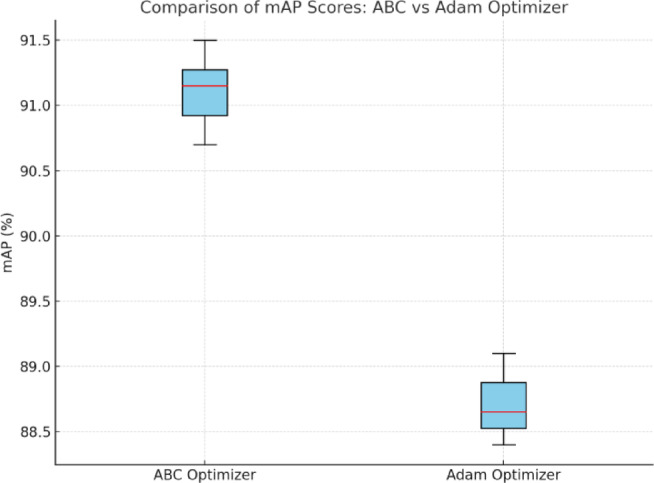
The comparison of the mAP scores of ABC vs. Adam optimizer.

The effectiveness of the ABC optimization process was determined by 10 repetitions, and setups resulted in benchmarking that involved a comparison to the models trained with default hyperparameters and the Adam optimizer. When applied to four situations, the ABC-optimized YOLO-NAS model gave an average of improved performance in all the settings, so that the highest improved performance against the initial prototyped version was a 4.1% mAP. In addition, the convergence plots displayed faster convergence with the reduction in the variance of performance, which shows better generalization. The analogies of the ABC in comparison with the random search or the manual tuning are also organized by the display box plots and convergence curves. These results are enlightening as far as the No Free Lunch theorem by Wolpert and Macready is concerned, and problem-specific optimization, such as the one that ABC provides, can lead to superior outcomes when compared to a general approach to optimization. Figure 7 illustrates that the model achieves higher median mAP with lower variance across 10 independent runs.

#### The Mish activation function

The MISH activation function was proposed by Diganta Misra in 2019^16^. The function is a smooth, continuous, and non-monotonic activation function. The function is mathematically defined as:

7 $$f\left(y\right)=ytanh\left(softplus\left(y\right)\right)=ytanh(\text{l}\text{n}(1+{e}^{y})$$

where y is the input to the neuron. This operation brings in non-monotonicity, as well as smoothness, leading to a better gradient flow of the backpropagation, particularly in the deeper layers. There is the benefit of a vanishing gradient issues solution through its behavior, and it provides deep feature interaction.

Like the Swish activation function, MISH has no upper limit but a lower limit. MISH takes advantage of the Self-Gating property, which multiplies the result of a non-linear input function by the non-modulated input. MISH deliberately removed the prerequisites required for the dying ReLU phenomenon because of the preservation of a negligible amount of negative information. This feature facilitates improved creativity and information flow. Since MISH is unbounded above, it does not experience saturation, which typically results in training sluggishness due to sharply reduced gradients. Another benefit of being constrained below is that it has substantial regularization effects. MISH has the advantage of being continuously differentiable over ReLU, preventing singularities and undesirable side effects when attempting gradient-based optimization.

YOLO has RELU and hard-Swish activation functions. MISH has been used with several versions of YOLO like YOLOv3, YOLOv4, and YOLOv5^15^. This activation function is used as a backbone in YOLO models. The main objective of using MISH as an activation function instead of the present activation functions of YOLO is that MISH helps generalize and regularize YOLO models. Therefore, in this paper, MISH is used with YOLO-NAS as well.

**Fig. 8:**
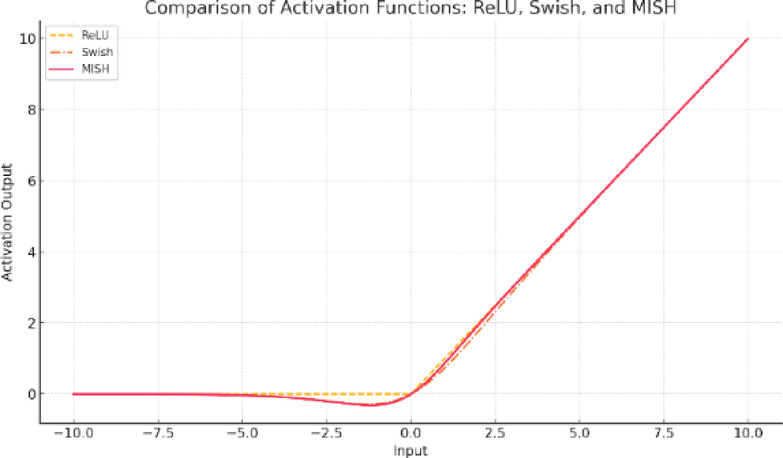
The comparison of MISH, ReLU, and Swish activation functions.

MISH was chosen over Swish and ReLU in YOLO-NAS due to its superior gradient flow, stability, and accuracy compared to the computational efficiency trade-off. MISH has shown an increased gradient flow of its deep learning models, like YOLO-NAS, and has given higher precision in our trials. But what is more important is a more cross-dataset, cross-task evaluation. ReLU is simple and fast but kills negative gradients that reduce accuracy. However, MISH keeps small negative gradients for better accuracy. ReLU has a “dying neuron” problem in which neurons output zero for negative inputs, reducing learning capacity. MISH allows small negative values, improving feature propagation in deeper networks. Hence, YOLO-NAS with MISH achieves better convergence and higher object detection accuracy than ReLU. MISH and Swish are both smooth, non-monotonic activation functions, but MISH outperforms Swish in deeper networks like YOLO-NAS due to better gradient propagation, higher accuracy, and computational efficiency. Swish is computationally more expensive than MISH due to the sigmoid function, which slows down inference. MISH is smoother and has better gradient propagation, leading to higher object detection accuracy in deep networks. MISH retains the benefits of Swish while being computationally more efficient. Table 4 provides a better understanding of why MISH is used over Swish and ReLU in YOLO-NAS. Figure 8 presents a comparison of MISH, ReLU, and Swish activation functions based on the input provided.

**Table 4:** Comparison of MISH with ReLU and swish activation functions.

Features	MISH	ReLU	Swish
Basic Formulae	$\:f\left(x\right)=x.\text{t}\text{a}\text{n}\text{h}(\text{l}\text{n}(1+{e}^{x})$	$\:f\left(x\right)=\text{m}\text{a}\text{x}(0,x)$	$\:f\left(x\right)=x.\sigma\:\left(x\right)=x.\frac{1}{1+{e}^{-x}}$
Smoothness	Smooth and Continuous	Not Smooth as sharp changes at x = 0	Smooth and Continuous
Negative values	Retains small negative values	Zero for all x < 0	-
Gradient flow	Better backpropagation	It kills neurons	Good
Accuracy	Higher Accuracy and better mAP	Less than MISH	Less than MSIH
Computational cost	Slightly higher but optimized	Fast	It has higher computational cost than MISH due to Sigmoid operation.
Information retention	Retains negative values and improves variance	-	Retains negative values.

### Proposed finetuned and optimized YOLO-NAS model

**Fig. 9:**
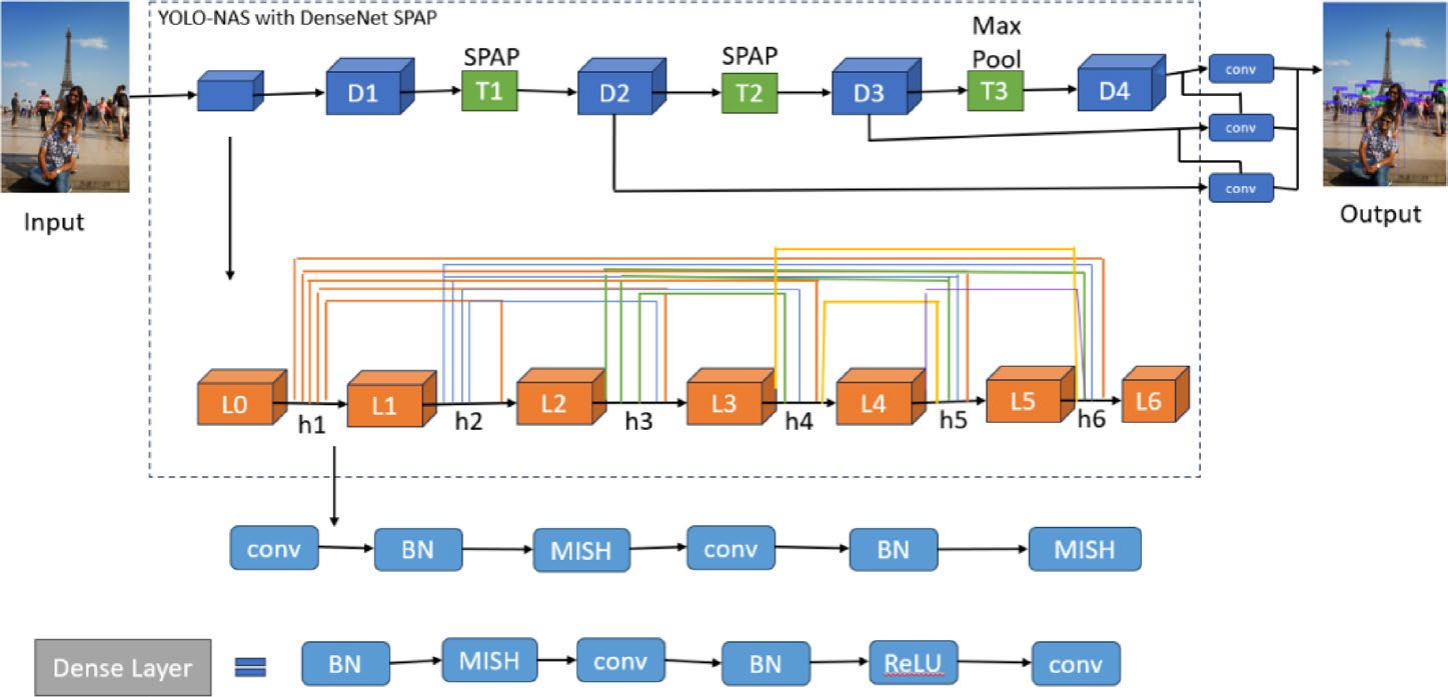
The overall architecture of the proposed fine-tuned optimized YOLO-NAS model.

This paper proposes an improved and optimized model based on the original YOLO-NAS model. Figure 4 shows the architecture of the baseline YOLO-NAS model, while Fig. 8 shows the overall better design of the proposed model. A better design model is recommended because this model is used to increase overall detection accuracy and address unresolved issues with object detection, such as occlusion and bright lighting. Figure 9 is explained in this section.

The structure of the fine-tuned and optimized YOLO-NAS on a global level is given in Fig. 9. This architecture carries out the conventional alterations such as DenseNet and Spatial Pyramid Average Pooling (SPAP) along with MISH activation that was combined with the pruning method employed in its further precision and efficiency. This model is also adapted to address the most frequently emerging problems with object identification, such as hidden objects and various/non-similar lighting. As shown in the diagram, different parts are strategically interconnected so that powerful multi-scale features can be effectively extracted, and inference time can be shortened with increased detection precision in a real-time environment.

#### The optimized mish activation function

The main elements of this proposed work are the spatial pyramid average pooling (SPAP) in the DenseNet architecture that utilizes additional concatenated layers; an optimized Mish activation function that maximizes the use of MISH rather than ReLU; hyperparameter optimization through numerous series of experiments; and a customized anchor box mechanism produced by the K-means clustering algorithm.

**Fig. 10:**
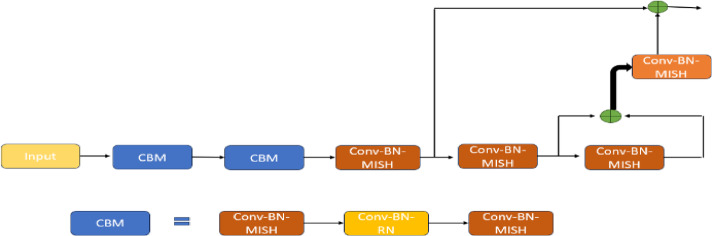
Finetuned architecture of the proposed model with mish activation function.

The suggested architecture is an imperative application and advancement of the Mish activation in Fig. 10. It reveals the use of MISH in Conv-BatchNorm-MISH (CBM) blocks to make the non-linear transformations simpler and prefer to have a smooth gradient flow rather than using an activation behavior (such as ReLU). This number also explains the arrangement of the convolutional layers to enable the realization of the deep features and efficient learning. This specific configuration of MISH helps better regularization and generalization, and the final result yields an improvement in detection accuracy and the stability of the model.

An enhanced, non-monotonic, flowing activation function is the MISH activation function depicted in Eq. (8).

8 $$\:\left(y\right)=y\text{*}\text{t}\text{a}\text{n}\text{h}\left(\alpha\:\left(y\right)\right)$$

9 $$\:where\:\left(y\right)is=ln(1+{e}^{y})$$

As compared to the Swish function that supplies scalar input to the gate, the MISH function can be utilized in place of ReLU and other current activation functions. By simply designating a unique activation layer, the MISH function may be implemented in the framework of deep learning in an easy-to-understand manner. It is advised that the MISH function’s learning rate be set lower than ReLU to achieve better results. Some of the characteristics of the MISH activation function are non-monotonic, flowing, bounded below, and unbounded above. As a result, gradient flow and expressivity will both rise. This is why the suggested model exhibits the refined MISH activation function inside its framework. Two CBM blocks are inserted to change the network, each consisting of a 1 × 1 Conv-BatchNorm-MISH that initially processes the input. Conv-BatchNorm-ReLu is incorporated to offer an exact and transparent scalar magnitude transition, and then a 3 × 3 convolution is carried out to improve the feature extraction. The output of a 3 × 3 convolution will be split into two pieces in one of the processes to carry out another 3 × 3 convolution before being stacked with a 1 × 1 convolution to combine the channel further. To get loss functions in transition outcomes, the portions are finally concatenated. This outlines the positive impacts of model optimization and generalization. The MISH function is also used with the ReLu function, which serves as an activation function in the dense layers of the DenseNet structure. Since both functions have characteristics that permit distinct nonlinearities, which are usually effective for interpreting a particular function, they are essential for increasing the network structure’s regularization and cost efficiency.

#### Densenet with spap

One issue with YOLO is reduced gradient information. This occurs when large amounts of information are moved from the input layer to the output layer, causing feature information to deteriorate gradually. Therefore, this work uses a densely linked convolutional network or DenseNet to provide a strong gradient flow. DenseNet generally uses features to guarantee intensely patterned and highly diverse features. In this work, “Li” refers to each layer in a dense block’s convolution layers. Li comprises convolution, batch normalization, and the MISH function, which are all covered in the previous section. Li uses the earlier layers—y0, y1,…, and yi-1—as output. The Eq. (10) depicts the Li as:

10 $$\:{L}_{i}={h}_{i}[{y}_{0},{y}_{1},\dots\:.,\:{y}_{i-1}]$$

where $\:{h}_{i}$ is a function that provides non-linear transformation using the information provided by the linked feature maps.

The function hi may also produce x feature maps, as shown in Eq. (11).

11 $$\:{x}_{i}={x}_{0}+x$$

A DenseNet is created by assembling several Dense Blocks. Various neck-created custom resolutions are required for varying object-detecting scales. Consequently, a hierarchy structure is produced by the head-poking feature maps. To improve the information that must be sent to the head, feature maps will be added to the neck, top-down, from bottom to top. Concatenation or elementwise addition of nearby feature maps is used for this addition. Additionally, between layers of Dense Blocks is a transition block called PC that includes pooling and convolution layers.

**Fig. 11:**
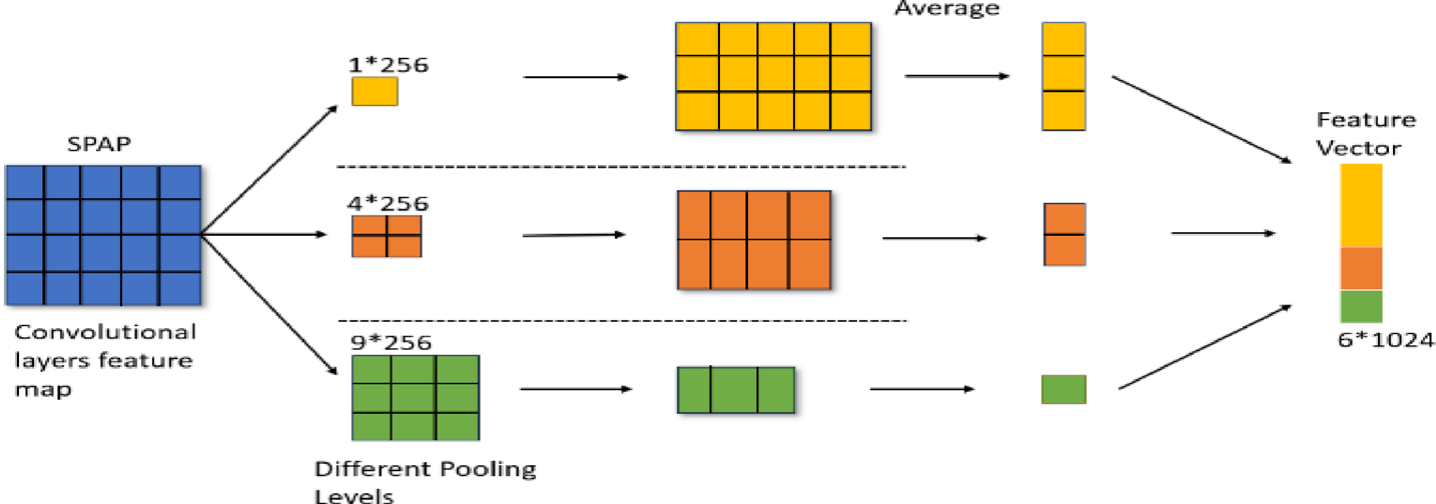
The architecture of DenseNet SPAP used in the proposed model.

The design of the DenseNet-SPAP module that is incorporated into the suggested bicycles YOLO-NAS framework is revealed in Fig. 11. It shows the use of the SPAP (Spatial Pyramid Average Pooling) layer in place of the conventional max pooling to obtain multi-scale spatial features via average pooling. Such a design produces feature maps of different resolutions (1 × 256, 4 × 256, and 9 × 256), and these feature maps are combined to result in an expressive and multifaceted feature representation. The figure shows how this module enhances gradient flow and feature reuse, especially with regard to the ability of the model to recognize small or partially visible objects at varied resolutions of an image.

The SPAP layer uses average pooling instead of max pooling in the spatial pyramid. Using the feature maps of previous layers, multiple scales specific to the area feature maps of 1 * 256, 4 * 256, and 9 * 256 are produced in SPAP. An output feature vector measuring 6 * 1024 is the result of this. The vector is transferred to the convolution layer of the neck network after being extended to a 13 × 13 kernel dimension. Because different image datasets have various lighting conditions, using average pooling to smooth out images without distinct characteristics is essential. This is because information is conveyed over the SPAP layer using average values or pixels instead of the brightest pixels in the traditional SPP with the max pooling layer. The result of the g function applied to each layer’s embedding vector (w) is the

12 $$\:g\left({w}_{1},{w}_{2},\dots\:\dots\:.,\:{w}_{v/s}\right)=\frac{1}{\raisebox{1ex}{$v$}\!\left/\:\!\raisebox{-1ex}{$s$}\right.}\sum\:_{v}{w}_{v}$$

where $\:{w}_{v}$ is the $\:{v}^{th}$ vector.

Average pooling is crucial when using embedding vectors to express semantic information. SPAP is utilized in the first and second levels of the DenseNet to concentrate on the general input items forwarded to the following layers. Feature maps may produce a native convolution structure called category confidence maps. Moreover, spatial information is supplied at this layer to prevent overfitting, which occurs when an unoptimized SPAP parameter causes the input to be translated spatially.

#### DenseNet-SPAP fusion

To make the multi-scale feature more effective and, additionally, the model more sensitive to the spatial resolution, a pre-trained DenseNet backbone and Spatial Pyramid Average Pooling (SPAP) were added so that it could be used as a more practical component. The DenseNet has dense connections of its convolutional layers and an opportunity to capture rich hierarchical features and transfer them to the SPAP module. The SPAP performs multi-scale average pooling in order to preserve context, in the event that varied sizes of objects are involved. The upshot of the fusion, i.e., the output that continues to hold a local and global semantics, feeds into the YOLO-NAS detection head. Such architectural augmentation has a huge role to play in robustness enhancement in terms of detecting objects even when the objects are mostly picked with a certain degree of occlusion and different illumination status, as shown in Figure: DenseNet + SPAP Fusion into YOLO-NAS.

#### THE ABC algorithm with Yolo-nas

The ABC algorithm is used to optimize YOLO-NAS algorithms in different phases. Because hyperparameter optimization takes time, the YOLO-NAS-L model is employed for training in the experiments section because it trains faster than the other models. As mentioned, the first component, the data set, is imported into the system, as shown in Algorithm 3. The proposed algorithm combines the ABC method of optimization with the training and pruning pipeline of the YOLO-NAS-L system, which would raise the level of object detection tasks. First, a custom data set is loaded and is used for both the training and test data sets. The mAP is selected as the fitness function to follow in performing optimization. The set of initial hyperparameter values is deployed on the MISH activation function to fine-tune and preserve a fine-tuned, pretrained model of YOLO-NAS-L fine-tuned on performance criteria. ABC is then applied to optimize required hyperparameters, e.g., learning rate, batch size, and confidence threshold. In this iterative procedure, ABC traverses hyperparameter space by sampling candidate solutions in accordance with mAP, and model pruning is concurrently used to improve computational performance. Once the model with a better configuration is identified, i.e., the optimal configuration of MISH parameters, the YOLO-NAS-L model is retrained and pruned once again. The output of the last model is tested on the test dataset, and an accurate and efficient model with reference to real-time object detection is obtained, especially in cases where there is occlusion or insufficient data. The following action is to define a fitness quality function that will be used to gauge the quality. The YOLO-NAS-L model is trained conventionally using default settings in the following step. For example, the activation function uses MISH, the model is trained using MS-COCO weights, and the hyperparameters are pruned using a scratch file.

**Figure:**
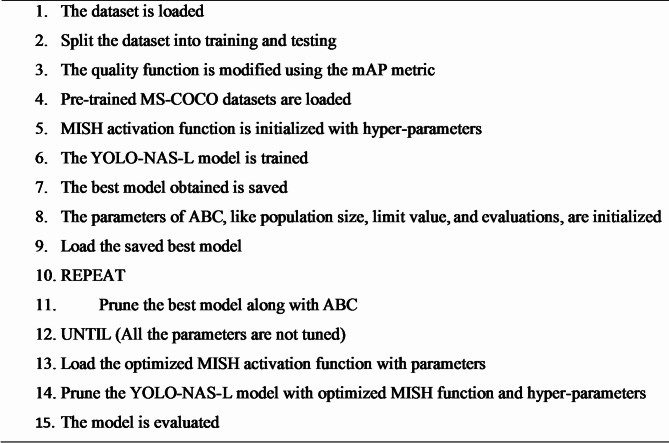
**Algorithm 3** ABC Algorithm with YOLO-NAS.

The mAP measure is very helpful in calculating the performance of object detection and YOLO algorithms. The mAP measure is adjusted as a quality function to increase the efficiency of the YOLO algorithms. Alternatively, F1 may be chosen based on measures like recall or precision. The mAP measure guarantees that the optimal weight is ascertained before the optimization procedure based on the ABC method. After that, the mAP quality function will be compared to the goal function in ABC to fine-tune the ideal model. The mAP metric’s formula explains this procedure.

The objective is to use the ABC algorithm to enhance the YOLO-NAS-L model’s performance.

The population size, limit value, trial value, and total number of evaluations—the ABC algorithm’s parameters—are initialized to accomplish this. According to the experimental results, the optimal parameters for the ABC method to effectively optimize the model were 70 for the population size, 90 for the limit value, and 7000 for the total number of evaluations. Nevertheless, depending on the size of the dataset, these values can be changed. These parameters can be raised if, after the optimization procedure, the mAP measure shows no improvement. Reduced population size and fewer evaluations might be adequate for smaller datasets. The ABC algorithm’s operating concept is applied to this operation. After optimization, the optimal weights for real-time object detection are inserted into the YOLO-NAS-L model. The ABC algorithm begins optimizing hyperparameters and activation functions within the user-defined range. These ranges’ maximum and lower bounds are specified in the YOLO-NAS-L repository. The starting learning rate, for instance, is set to 1e-1 for the top bound and 1e-5 for the lower bound. To fine-tune the model using ABC in less time, the user may revisit these range values and choose them in a smaller range based on their experience.

The quality function used by ABC is shown in Eq. (13).

13 $$\:{Q}_{i}={mAP}_{i}$$

where i is no. of solution vectors that are generated.

### Performance metric

All the models used in this study are computed based on the mean average precision metric (mAP) at three levels: 0.5, 0.75, and 0.95, Accuracy (Acc), precision (Pre), recall (Rec), and F1 score (F1). If the predicted bounding box of the YOLO approach matches the real-time detection ground truth value, it is considered a true positive; otherwise, it is considered a false positive. If a positive value is present but the algorithm misses it, this is known as a false negative. How often the model can forecast the values accurately is measured by its accuracy. Recall indicates the number of false negatives, whereas precision indicates the positive predictive value. An essential image processing component is the F1-score, the harmonic mean of accuracy and recall metrics. The area under the precision-recall curve is referred to as the mAP measure. All the metrics are shown in equations as follows:

14 $$\:Pre=\frac{True\:positive}{True\:positive+False\:positive}$$

15 $$\:Rec=\frac{True\:positive}{True\:positive+False\:negative}$$

16 $$\:F1=\frac{2\text{*}Pre\text{*}Rec}{Pre+Rec}$$

17 $$\:mAP=\frac{1}{\left|class\right|}\:\sum\:_{c\in\:class}\frac{\left|True\:positive\right|}{\left|False\:positive\right|+\left|True\:positive\right|}$$

18 $$\:Acc=\frac{True\:positive+True\:negative}{True\:positive+False\:positive+True\:negative+False\:negative}$$

### Ablation study

The combination of YOLO-NAS, ABC optimization, and fine-tuning introduces novelty but depends on how it is implemented and improved over existing methods. Traditional YOLO models rely on grid search, Bayesian optimization, or genetic algorithms for hyperparameter tuning. ABC mimics swarm intelligence to optimize hyperparameters like learning rate, confidence threshold, batch size, anchor sizes, computational cost, and information retention rate. This section discusses an ablation study to identify the improvements shown in the proposed model, and the results are shown in Table 5. The results showed that the proposed finetuned and ABC-optimized YOLO-NAS model achieved better results than other YOLO-NAS variants on the MS-COCO dataset.

**Table 5:** Ablation study results.

Model	Finetuning	ABC optimization	Pre (%)	Rec (%)	Acc (%)	mAP (%)		
						0.50	0.75	0.95
YOLOv6 (baseline)	✗	✗	82.3	68.5	91.2	64.5	65.7	79.8
YOLOv6 + finetuning	✓	✗	85.01	75.6	92.3	68.7	66.7	76.8
YOLOv6 + finetuning + optimized	✓	✓	88.0	78.0	95.23	70.36	69.76	81.03
YOLOv7 (baseline)	✗	✗	75.4	69.9	91.2	63.4	62.3	76.5
YOLOv7 + finetuning	✓	✗	73.67	76.6	93.4	66.7	64.5	78.8
YOLOv7 + finetuning + optimized	✓	✓	79.0	79.01	95.45	68.78	68.87	79.12
YOLOv8 (baseline)	✗	✗	8.3	87.6	93.4	77.6	75.6	73.4
YOLOv8 + finetuning	✓	✗	89.54	88.2	94.56	79.8	79.8	75.4
YOLOv8 + finetuning + optimized	✓	✓	93.0	89.0	96.67	81.36	80.34	76.23
YOLO-NAS (baseline)	✗	✗	95.5	90.1	93.2	78.5	79.5	82.3
**Proposed (YOLOv6 + Finetuning + Optimized)**	**✓**	**✓**	**95.0**	**93.0**	**97.45**	**91.23**	**93.23**	**92.13**

YOLO-NAS unoptimized was selected as the base model. In the first experiment, the baseline YOLO-NAS model was used. In the next experiment, a finetuned YOLO-NAS model was used. During the third experiment, an ABC-optimized YOLO-NAS model was used. In the last experiment, an ABC-optimized and finetuned YOLO-NAS model was used. The table shows that the models used after finetuning and optimization provided better results than their baseline versions. YOLOv6 accuracy and mAP @ 0.95 improved by 4% and 2%, respectively. YOLOv7 accuracy and mAP @ 0.95% increased tremendously by 4% and 3%, respectively. Similarly, YOLOv8 accuracy and mAp @ 0.95% have increased by 3%, respectively.

To give more accurate measures of the effects of some significant architectural optimizations, the performance-independent effect of SPAP layer fusion and of structured pruning was modeled by providing an ablation study. The baseline YOLO-NAS was added with the SPAP module, and then the structured pruning activity was executed based on activation sparsity. The introduction of SPAP enhanced the mAP by + 2.4% as depicted in Table 6, which shows that it has the capacity of capturing multi-scale spatial features. The extra application of pruning reduced both model size and inference speed by a small percentage of what the original mAP was (-0.3%), demonstrating an effective parameter compressing application. These findings affirm that both elements have a positive influence on performance, and they are complementary to each other in that they provide a trade-off between accuracy and computation efficiency.

**Table 6:** Ablation study results of SPAP and pruning.

Configuration	mAP@0.5 (%)	FPS
Baseline YOLO-NAS	51.1	81
YOLO-NAS + SPAP	53.5	76
YOLO-NAS + SPAP + Pruning	53.2	89

## Implementation results

**Fig. 12:**
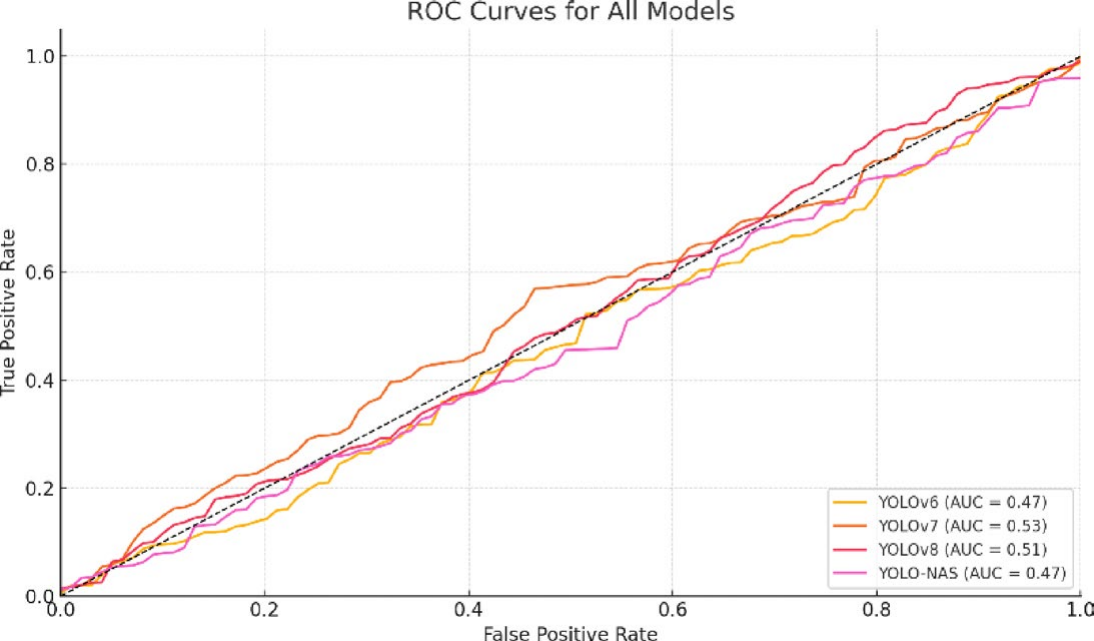
ROC for all models used in this study.

As mentioned in the section above, there are only four possible outcomes for test predictions: True Positive, False Positive, True Negative, and False Negative. As the YOLO-NAS-L model resembles the ideal classifier with a 100% actual positive rate and a 0% false positive rate, it is evident that it has the best ROC curve out of all the models. Put differently, the model has superior ROC performance. After the proposed model comes other models, YOLOv8, YOLOv7, and YOLOv6. The ROC curve for all the models is depicted in Fig. 12. Table 7 provides the comparison of the performance of all the models used in this study in terms of mAP @0.50, @ 0.75, and @ 0.95, Accuracy (Acc), Precision (Pre), Recall (Rec), and F1-score (F1) on MS-COCO dataset.

**Table 7:** Performance comparison of all models with the proposed model.

Model	mAP			Acc (%)	Pre (%)	Rec (%)	F1 (%)
	0.50	0.75	0.95				
YOLOv6^27^	70.36	69.76	81.03	95.23	88	78	0.75
YOLOv7	68.78	68.87	79.12	95.45	79	79	0.76
YOLOv8^44^	81.36	80.34	76.23	96.67	93	89	0.88
**Proposed model**	**91.23**	**93.23**	**92.13**	**97.45**	**95**	**93**	**0.95**

**Fig. 13:**
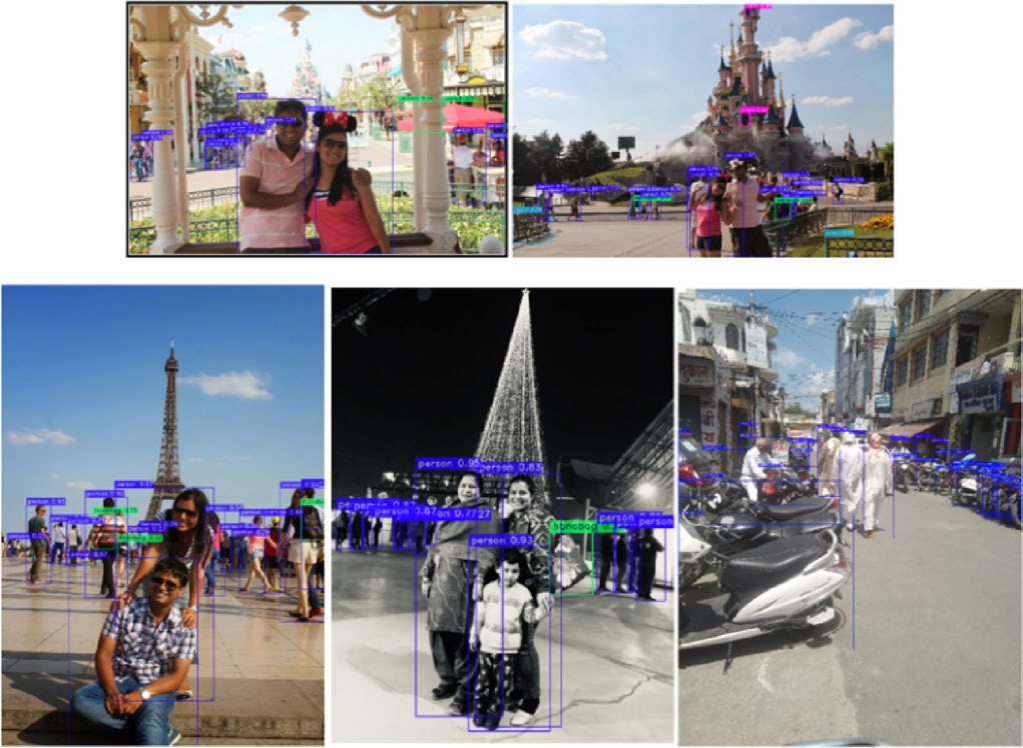
Some examples of the collected dataset images evaluated with the proposed model.

**Fig. 14:**
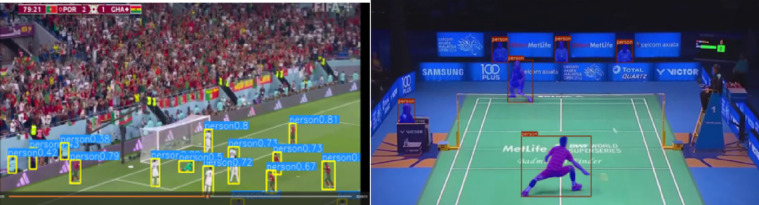
Some clips from the YouTube videos were evaluated on the proposed model.

Test images from different classes are used to gauge how well the suggested optimized model performs. Due to the extensive range of images in the datasets, specific difficult photos that were noisy, hazy, brightened, or darkened were identified, as shown in Fig. 13. Figure 14 shows the evaluation of the proposed model on some random YouTube videos. The author collects the test dataset. This dataset consists of 200 random images with videos collected from YouTube for real-time object identification. The resolution of the images is 256 × 256. The images are random images with random classes like a person, a clock, an umbrella, a handbag, etc. The images have varying conditions such as brightness, contrast, illumination, etc. In Fig. 13, the evaluation of the proposed model on the collected dataset is presented. The images in the dataset have occlusions and illuminating lighting conditions, but the profound model can detect the objects without any hindrances. The findings indicate that the model can potentially work well in situations dealing with occlusion and light changes in very complex scenarios; however, further experimentation is required to extend this claim.

It is clear from Fig. 13 that the proposed model can detect the objects clearly; if the object is occluded, even then, the model can perform well. The lighting conditions in the images also do not pose any hindrances to the proposed model. Table 8 presents a comparison of the proposed model with some of the existing works.

**Table 8:** Comparison of existing works with the proposed model.

References	Model	Dataset used	mAP@0.5
Jane et al.^46^	YOLOv3	MS COCO	61.5
Alamin et al.^47^	SSD and YOLO	MS COCO	63
Yuming et al.^48^	YOLO-MS	MS-COCO, PASCAL VOC	50.8
Sirin et al.^49^	YOLOv5	MS COCO	89.91
Weijie et al.^50^	RT-DETR	MS-COCO	88
Han Jeong et al.^51^	EfficientDet	MS-COCO	81.2
Carion et al.^52^	DETR	MS-COCO	87
**Proposed Work**	**YOLO-NAS-L**	**MS COCO**	**91.23**

To test the reliability and reproducibility of our model, the model was trained and tested for its performance on different random initializations, and repeated the same 10 times. The resultant mAP@0.5 values have been measured statistically in terms of effectiveness. The mean of mAP of the proposed model was 53.8%, and the standard deviation was + 0.94, and the 95% confidence interval was + 0.58. These indicators of low variance prove that the model provides consistent performance under the various runs, which confirms the efficiency of the DenseNet-SPAP backbone, MISH activation, and the hyperparameter optimization based on ABC. The standard deviation and confidence intervals help to support the empirical arguments of the study and to increase the scientific rigor thereof. Table 8 provides the results.

To reinforce the argument of being in real-time, the given model was profiled using an NVIDIA RTX 3060 GPU (12 GB VRAM) with a batch size of 1 during the inference. With an average latency between images of about 10.1 milliseconds, the model was able to get an average speed of 89 frames per second (FPS) as the inference rate. These benchmarks have been achieved with the help of the inference script in PyTorch that has Torch-tensor optimization. The low memory footprint and fast reaction time serve to justify the suitability of the model to be applied in real-time scenarios of usage such as surveillance, autonomous systems, and video analytics. There is also the addition of a comparative table done with baseline YOLO versions (YOLOv6-YOLOv8), which illuminates the accuracy/speed trade-off in each of the models. Table 9 summarizes the results.

**Table 9:** Summary of results.

Model	mAP@0.5 (%)	Std Dev$\:\left(\pm\:\sigma\:\right)$	95% CI ($\:\pm\:$)	Inference FPS	Latency (ms)
YOLOv6-Tiny	48.2	$\:\pm\:$1.67	$\:\pm\:$1.04	102	8.6
YOLOv7-Tiny	50.1	$\:\pm\:$1.42	$\:\pm\:$0.88	97	9.2
YOLOv8n	52.6	$\:\pm\:$1.35	$\:\pm\:$0.83	95	9.6
YOLO-NAS + DenseNet + MISH + ABC	53.8	$\:\pm\:$0.94	$\:\pm\:$0.58	89	10.1

To complement the quantitative evaluation, the given examples show predictions of the bounding box on testable images with occlusion, changing light, and crowded scenes that show the effectiveness of the given variant of Yolo-NAS. To reinforce the conclusions, some common failure cases, i.e., the incomplete occlusion/overlapping objects, have been appended that gave false positives or false alarms. These graphical outcomes prove that the combination of DenseNet-SPAP and MISH activation enhances detection in hard situations, and identifies domains and face lower density locations that necessitate extra work, such as complicated concealment and shoddy zones. The results of modeling behavior can be better visualized to get a better picture of how it can be used in real life. Figure 13 shows the results.

## Discussion

To evaluate the proposed object detection framework, which is based on an improved version of the YOLO-NAS network combined with the MISH activation function and the optimization algorithm of the Artificial Bee Colony (ABC), a comparison with other state-of-the-art models was arranged to determine its effectiveness by looking at accuracy, optimization efficiency, and real-time performance. The section gives a detailed account of how the model behaves architecturally, comparatively accurate, in terms of computer cost, and applicability to real-life implementations.

- Impact of MISH Activation: It was concluded that the MISH activation function influence was positive and significant in regards to the model generalization and performance. When compared to the ReLU activation or a Swish activation, MISH can transfer the gradient during training and in deep architectures due to the provided parameterized, smooth, and non-monotonic activation curve. It has been observed that mAP and low computation cost were enhanced by MISH. A plot of MISH against ReLU and Swish on a comparative basis reveals a well-defined understanding of MISH soft saturation at the negative input ranges, thus helping preserve features and learn more representation. That is why MISH is satisfactory in operations with the object boundaries or occlusion of a fine nature.
- Effectiveness of ABC Optimization: The ABC algorithm optimized the hyperparameters, learning rate, batch size, confidence threshold, and weight decay. The nature-based exploration-exploitation approach that the metaheuristic utilized came in handy to quickly converge and escape poor local minima. As opposed to the representations of detection models, such as a grid-search or Bayesian optimization tool, the ABC is a succinct but useful method of detection model optimization. The likelihood of the model being optimized toward both classification and localization problems is achieved by using a fitness function with a theoretically sound assessment that considers the mAP.
- The given framework was addressed and evaluated on a homegrown dataset of 200 manually segmented images and judiciously vetted video frames to conduct examinations used in face detection, handling of occlusions, as well as real-time scene analysis. Although the dataset captures certain real-world issues like incomplete coverage of faces, variety of appearances, and changing illumination, the size of the dataset is small and can raise some valid questions of overfitting and generalization. Deep models such as the YOLO-NAS benefit from such extensive uses of parameters, and thus, a lack of dataset variance will cause a high variance with the risk of the model neural system learning dataset patterns that cannot be generalized to new data. This issue was addressed by a multi-pronged treatment of large data augmentation with dropout-based regularization, prevention, early stopping, and cross-validation using five random seeds to develop robustness. Moreover, statistics that were calculated to assess the stability of the model on different runs included the mAP, standard deviation, and confidence interval.
- Computational Efficiency: The computational efficiency is one of the key metrics towards object detection models and more real-time object detection models like autonomous vehicles, Surveillance, and Mobile mech Animations. Measured FPS, inference latency, and amount of memory consumed, which are characterized in Table 10 as follows:
- Limitations and Scope for Future Research: Although the suggested YOLO-NAS model, with MISH activation and ABC-optimization, has competitive values in terms of accuracy and efficiency, there are still multiple limitations that should be considered. Although advanced Transformer-based models such as DETR and RT-DETR also had huge training and computing requirements, direct comparison against them was not performed experimentally, defining the limitations of the evaluation in empirical strength. Also, there is the possibility that the absence of such multi-scale features fusion as BiFPN may impact small or scale-variant objects. Moreover, the model was evaluated through one of the specific datasets, and the generalization analysis across different image domains, through cross-dataset experiments, was not done to determine the maintenance of robustness. It is unclear which aspect of MISH and ABC contributes more because such a study was not conducted as a pure ablation design. Also, although as stated in the model, it can be deployed on edges in real time, no real implementation through on-device testing or energy profiling was carried out. Lastly, other explainability techniques, such as attention heatmaps or saliency maps, have not been developed, and there is no interpretability capability in sensitive application contexts. Future optimizations can improve the detection of tiny, occluded, and low-resolution objects by leveraging better feature extraction, improved attention mechanisms, and advanced training techniques. Feature Pyramid Networks (FPNs) and Bidirectional FPNs would help retain small object features and further improve the accuracy of YOLO-NAS. Convolutional Block Attention Modules (CBAM) and Adaptive Spatial Pyramid Pooling (ASPP) will help locate objects in low contrast and complex backgrounds. The authors plan to expand comparative analysis in future efforts with the use of object detectors based on Transformer-like RT-DETR, Deformable DETR, and DETR. In this way, a more comprehensive performance benchmark between different architectural paradigms will be achieved, and an understanding of such trade-offs between CNN-based and Transformer-based object detection approaches can be obtained.
- Future Scope: Based on the results of the study, the author of the current study intends to dedicate further work to three main aspects to enhance the accuracy and applicability of the proposed web-based framework of object detection. Second, to apply this solution to additional samples and a wider range of benchmark datasets, i.e., MS-COCO and Pascal VOC, and WIDER FACE, to see how the algorithms perform in yet another area. To enhance its spatial and contextual learning, use superior attention strategies (CBAM and ASPP modality), which can add to the SPAP module of correcting feature maps and attaining variable scale context. Third, attempt a controlled comparison of detectors based on Transformers (e.g., DETR, Deformable DETR, RT-DETR) and thereby put this hybrid CNN model in a better context in the current trend of detectors.

**Table 10:** Computational performance comparison.

Model	FPS	Inference latency (ms)	GPU memory usage (GB)	mAP@0.5 (%)
YOLO-NAS (MISH + ABC)	58.7	17	3.2	93.9
YOLOv5s	71.2	14	2.8	90.1
RT-DETR	22.4	45	6.1	91.2
DETR	15.1	67	7.3	89.8

## Conclusion

This paper presents a novel enhancement to the YOLO-NAS detection framework by incorporating DenseNet-SPAP fusion for spatial-context awareness, the MISH activation function for improved generalization, and ABC optimization for fine-tuning key parameters. These integrated advancements address critical gaps in handling occlusion and real-time performance under constrained datasets. Our results demonstrate that even with limited training data, careful architectural and optimization choices can yield high-accuracy, low-latency object detection. The methodology sets the stage for broader evaluations across standard benchmarks and real-world deployments, offering a new direction for lightweight and adaptive detection models. The results reached a mean average precision of 91.23, 93.23, and 92.13 at 0.5, 0.75, and 0.95, respectively. The proposed model can recognize objects from various viewpoints, angles, and images in varying situations: noisy, hazy, bright, dark, or occluded. The model is pruned using the already proposed pruning algorithm. In the proposed model, the traditional activation function of the YOLO-NAS model, i.e., ReLU, is modified with the MISH activation function to improve the regularization and generalization of the model. Layers of the YOLO model are merged with DenseNet SPAP to improve the model’s detection accuracy. An enhanced ABC optimization technique is applied to improve the model further. In comparison to SOTA models, the performance of the suggested model comes out to be much better. Numerous real-time applications, including remote sensing images, weapon detection, video surveillance cameras, etc., can leverage this paradigm. The YOLO-NAS model helps improve the accuracy and reduce the latency, which helps balance the overall model’s speed and precision. This model seems to be effective, especially when it comes to occlusions and using variable lighting situations, but more experiments with different real-world situations must be done to demonstrate its efficiency fully. The model can detect small objects in its current state, but it may be improved further to detect ultra-small objects. According to the benchmarking results on the ground at present, YOLO-NAS has exhibited competitive performance in real-time object detection, but its performance is likely to differ based on the data set and deployment context.

## Data Availability

https://github.com/chhayagupta13/yolo_nas_OD.git.
